# Exploring the Association Between Residual Mood Symptoms and Self-Reported Side Effects in the Euthymic Phase of Bipolar Disorders: A Cross-Sectional Network Analysis

**DOI:** 10.1155/2024/3375145

**Published:** 2024-10-24

**Authors:** Nathan Vidal, Eric Brunet-Gouet, Solène Frileux, Valérie Aubin, Raoul Belzeaux, Philippe Courtet, Thierry D'Amato, Caroline Dubertret, Bruno Etain, Sebastien Gard, Emmanuel Haffen, Dominique Januel, Marion Leboyer, Antoine Lefrere, Pierre-Michel Llorca, Emeline Marlinge, Emilie Olié, Mircea Polosan, Raymund Schwan, Michel Walter, The FACE-BD (FondaMental Academic Centers of Expertise for Bipolar Disorders) group, Christine Passerieux, Paul Roux

**Affiliations:** ^1^Fondation FondaMental, Créteil, France; ^2^DisAP-DevPsy-CESP, INSERM UMR1018, Centre Hospitalier de Versailles; Service Hospitalo-Universitaire de Psychiatrie d'Adultes et d'Addictologie, Le Chesnay; Université Paris-Saclay, Université de Versailles Saint-Quentin-En-Yvelines, Villejuif, France; ^3^Pôle de Psychiatrie, Centre Hospitalier Princesse Grace, Pasteur, Monaco; ^4^CHU Montpellier, IGF, CNRS, INSERM, University of Montpellier, Montpellier, France; ^5^CHU Montpellier, Hôpital Lapeyronie, Psychiatric Emergency and Post Emergency Department, Pole Urgence, IGF, CNRS, INSERM, Université de Montpellier, Montpellier, France; ^6^INSERM, U1028, CNRS, UMR5292, Lyon Neuroscience Research Center, Psychiatric Disorders: From Resistance to Response Team, University Lyon 1, Villeurbanne, Lyon, France; ^7^AP-HP, Groupe Hospitalo-Universitaire AP-HP Nord, DMU ESPRIT, Service de Psychiatrie et Addictologie, Hôpital Louis Mourier, Colombes, Inserm UMR1266, Sorbonne Paris Cité, Faculté de Médecine, Université de Paris, Paris, France; ^8^Assistance Publique des Hôpitaux de Paris, Groupe Hospitalo-universitaire AP-HP Nord, DMU Neurosciences, Hôpital Fernand Widal, Département de Psychiatrie et de Médecine Addictologique, INSERM UMR-S, Université Paris Cité, 1144 Optimisation Thérapeutique en Neuropsychopharmacologie OTeN, Paris, France; ^9^Centre Hospitalier Charles Perrens, Pôle de Psychiatrie Générale et Universitaire, Bordeaux, France; ^10^Université de Franche-Comté, UMR INSERM 1322 LINC, Service de Psychiatrie de l'Adulte, CIC-1431 INSERM, CHU de Besançon, F-25000, Besançon, France; ^11^Unité de Recherche Clinique, EPS Ville-Evrard 93332, Neuilly-Sur-Marne, France; ^12^Univ Paris Est Créteil, INSERM U955, IMRB, Translational NeuroPsychiatry Laboratory; AP-HP, Hôpitaux Universitaires Henri Mondor, Département Médico-Universitaire de Psychiatrie et d'Addictologie (DMU IMPACT), Fédération Hospitalo-Universitaire de Médecine de Précision en Psychiatrie (FHU ADAPT), Créteil, France; ^13^Pôle de Psychiatrie, Assistance Publique Hôpitaux de Marseille, Marseille France, INT-UMR7289, CNRS Aix-Marseille Université, Marseille, France; ^14^Centre Hospitalier et Universitaire, Département de Psychiatrie, Université d'Auvergne, EA 7280, Clermont-Ferrand, France; ^15^Grenoble Institut Neurosciences, Université Grenoble Alpes, Inserm, U1216, CHU Grenoble Alpes, Grenoble, France; ^16^Centre Psychothérapique de Nancy, Inserm U1254, Université de Lorraine, Nancy, France; ^17^Service Hospitalo-Universitaire de Psychiatrie Générale et de Réhabilitation Psycho Sociale 29G01 et 29G02, CHRU de Brest, Hôpital de Bohars, Brest, France

**Keywords:** adverse drug effects, bipolar disorders, euthymic phase, mood symptoms, network analysis

## Abstract

**Introduction:** Bipolar disorders (BD) are characterized by mood symptoms that can worsen medication side effects. We aimed to study the association between residual mood signs and self-reported side effects in the euthymic phase of BD.

**Methods:** We assessed residual mood signs using the Montgomery–Asberg Depression Rating scale (MADRS) and Young Mania Rating scale (YMRS) and self-reported side effects using the Patient-Rated Inventory of Side Effects (PRISE-M) for 880 males and 1369 females with BD. We conducted a network analysis to test the associations between 52 items of the three scales for males and females separately. We then identified clusters of nodes that fit the networks well.

**Results:** We report only positive associations between residual mood signs and side effects. An elevated mood (YMRS) in females and increased energy (YMRS) in males were central nodes, strongly influencing the development of additional mood symptoms and side effects. Furthermore, we identified three clusters of nodes in both sexes: (1) a “mood cluster”, including most YMRS and MADRS items and the PRISE-M items evaluating sedation, sleep, and restlessness, (2) a cluster of nonsexual side effects (mostly PRISE-M items), and (3) a cluster of sexual side effects. In both sexes, we identified bridge nodes that may favor the communication between mood and side effects, namely palpitations (PRISE-M) and agitation (PRISE-M).

**Conclusions:** The results justify the particular attention of practitioners to monitor elevated moods or increased energy to try to reduce self-reported side effects and other residual mood symptoms in the euthymic phase of BD. Our findings suggest that clinicians could consider patient-reported loss of energy, difficulty in falling asleep, and restlessness as mood symptoms rather than medications' side effects. Palpitations and agitation may contribute to the development of additional mood symptoms or somatic complaints.

## 1. Introduction

Bipolar disorders (BD) affect ~0.4%–1.1% of the population [1] and are characterized by mood episodes, such as depression, hypomania, or mania, and frequent residual symptoms during euthymia. Between 49% and 70% of patients with BD in the euthymic phase continue to experience depressive (e.g., psychic anxiety) and manic symptoms (e.g., poor insight, sleep disturbances, and irritability), which are associated with higher risks of relapse [2]. Besides, residual symptoms can include sleep disturbance and cognitive impairment, which may be attributed to pharmacological treatments [3]. Indeed, the management of BD involves psychotropic medications that generally treat and prevent mood episodes, but may induce side effects, including asthenia, sedation, and weight gain [4, 5]. Residual symptoms can be interpreted as side effects, prompting the patient to stop the treatment, thus increasing the risk of relapse [6]. Besides, some medications' side effects, such as akathisia, are associated with more intense mood symptoms [7]. Overall, differentiating residual symptoms of depression or mania from medication side effects is a critical clinical challenge that can help prevent relapse.

It is important to consider sex effects on both symptom and side effect patterns. Distinct patterns of reported side effects have been reported for males and females with BD exposed to the same medication. For example, females with BD taking lithium tend to experience weight gain, whereas males are more inclined to report tremors [8]. In addition, subjective tolerance to antipsychotic-related adverse effects is lower for females than males [9], and females may exhibit higher drug plasma concentrations for the same antidepressant dose [10]. Moreover, some of the usual side effects of psychotropic drugs are sex-specific (i.e., irregular periods or erectile dysfunction) [11]. Beyond medication-related differences, there are sex-based differences in the expression of mood symptoms, with males showing significantly more intense depressive and manic residual symptoms than females [2]. Therefore, the association between subjective side effects and mood in BD is likely influenced by the sex of the individual.

Network analysis is appropriate to assess the association between patient-reported side effects and residual symptoms of BD because it estimates all associations, called edges, simultaneously between items, called nodes, without favoring predefined hypotheses [12]. It has been extensively used to study symptoms and comorbidities of mental disorders, suggesting pathways to comorbidity and identifying diagnostic boundaries [13–16]. Network analysis includes four tools that can offer insights into the association between patient-reported side effects and residual mood symptoms. First, the identification of central items in the network, based on centrality values [17], makes it possible to identify the items that are more likely to affect the rest of the network. Indeed, central symptoms have greater clinical relevance [18] and can help make predictions about recovery and the prognosis of mental disorders [13]. We hypothesized that “concentration problems”, “loss of energy”, and “feeling sad” would demonstrate relatively higher centrality than other items in our network, as reported in previous studies [19–21]. Second, predictability is an absolute indicator of the strength of the network estimation for each node [22] that can guide future interventions aiming to reduce side effects or residual symptoms [23]. Third, nodes can form clusters, groups of nodes more highly associated with one another than with the rest of the network, which can be detected by community-detection algorithms. Identifying clusters can be particularly helpful when assessing the relationship between various scales to detect unexpected groups of nodes that overlap the original constructs assessed by the items [13]. Fourth, investigating the positioning of the nodes within these clusters is crucial for understanding the relationships between them. The nodes that link two clusters are called bridge nodes (or “communicating nodes” [24]) and could contribute to the propagation from one cluster to another, indicating the emergence of comorbidity between clusters [25]. On the contrary, nodes at the center of a cluster are stabilizing nodes and could favor the development of the cluster, thus indicating the mechanisms leading to the chronicization of the cluster [24]. Identifying bridge and stabilizing nodes would make it possible to study the propagation and chronicization of mood and self-reported side effects in euthymic BD.

## 2. Methods

Our study adhered to the STROBE guidelines for observational study reporting proposed by the EQUATOR team [26] (Data S1).

### 2.1. Description of the Sample

We included euthymic patients recruited into the FACE-BD (FondaMental Advanced Centers of Expertise for Bipolar Disorders) cohort between February 2009 and February 2022 within a French national network of 12 centers (Bordeaux, Créteil, Montpellier, Grenoble, Nancy, Marseille, Paris, Versailles, Monaco, Clermont–Ferrand, Colombes, and Besançon). This network was created by the Fondation FondaMental (https://www.fondation-fondamental.org), which organized and provided the necessary resources to follow cohorts of patients with BD, promoting comparative research.

The diagnosis of BD was based on the structured clinical interview for DSM-IV-TR (SCID) criteria [27]. Outpatients between 18 and 65 years of age with type I, II, or not otherwise specified (NOS) BD were eligible for the present study. We excluded patients with missing data for the Montgomery–Asberg Depression Rating scale (MADRS), the Young Mania Rating scale (YMRS), or Patient-Rated Inventory of Side Effects (PRISE-M) questionnaires. Finally, we only included euthymic patients, defined as those with no characterized mood episode (depression, hypomania, or mixed episode) at the time of testing according to DSM-IV-R criteria and with a MADRS total score ≤10 and a YMRS total score ≤12 (i.e., common liberal thresholds) [28].

### 2.2. Measurements

We recorded the sex at birth of the patient (self-report), their age, their type of BD, the total number of mood episodes, the age at onset of BD, and the history of psychotic symptoms. We also reported the score of the Clinical Global Impressions (CGI) scale, which assesses symptom severity [29]. We ensured that our study was compatible with the Sex and Gender Equity in Research (SAGER) guidelines [30].

#### 2.2.1. Adverse Drug Effects

We assessed adverse drug effects using the modified PRISE-M [31]. The PRISE was originally developed within the framework of STAR*⁣*^*∗*^D, a multicenter clinical trial for outpatients with nonpsychotic major depressive disorders. STAR*⁣*^*∗*^D aimed to identify the most effective treatment options for individuals who did not satisfactorily respond to citalopram. They first developed the PRISE with eight items to assess potential adverse drug effects and help clinicians adapt the treatment of the 4000 participants with major depressive disorder. Twenty-four items were later added to the PRISE to constitute the PRISE-M [32]. The PRISE-M evaluates the level of tolerance for 32 side effects. We found good to excellent internal consistency of the questionnaire in euthymic males (McDonald's *ω* = 0.85) and females (McDonald's *ω* = 0.86). The 32 items are coded 0 for absent, 1 for bearable, and 2 for painful.

#### 2.2.2. Mood

We assessed the intensity of residual depressive symptoms using the MADRS [33]. The MADRS includes 10 items that evaluate a range of depressive symptoms, including suicidal ideation and feelings of sadness or inner tension. Each item is rated on a scale from 0 to 6, with higher scores indicating greater severity of symptoms. The MADRS demonstrated good interrater reliability (correlation coefficient > 0.89), good concurrent validity (Pearson's *r* > 0.81), good to excellent internal consistency (Cronbach's *α* = 0.85), and sensitivity to change [33, 34]. We measured low internal consistency of the MADRS in our sample (McDonald's *ω* = 0.46), probably due to the thresholding of MADRS at inclusion in the study.

We evaluated the intensity of residual manic symptoms using the YMRS [35]. The 11 items of the YMRS are rated by severity. Four items are graded from 0 to 8 (irritability, speech, thought content, and disruptive/aggressive behavior), while seven items are graded from 0 to 4. The YMRS showed excellent interrater reliability (correlation coefficient of 0.93 for the total score), good concurrent validity (correlation coefficient of 0.88 between the YMRS and an independent global rating), and demonstrated good sensitivity to change [35]. We measured good internal consistency of the YMRS in our sample (McDonald's *ω* = 0.68).

The MADRS and YMRS were administered by a trained clinician of the FACE-BD network and were based on patients subjective reports of their clinical condition during the past 48 h and the clinician's observations during the interview.

#### 2.2.3. Treatments

We recorded the presence/absence of several classes of treatment (antidepressants, anticonvulsants, lithium, antipsychotics, anxiolytics, and anticholinergics prescribed for extrapyramidal side effects). We report chlorpromazine equivalents (CPZeq, computed from the formulas proposed by [36, 37]) and the anticholinergic burden assessed using the scale designed by Salahudeen, Duffull, and Nishtala [38] (Data S2).

### 2.3. Network Analysis

We conducted network analysis with the items of the PRISE-M, MADRS, and YMRS to better characterize the relationships between residual mood symptoms and side effects in BD.

#### 2.3.1. Network Estimation

All analyses were conducted using R (version 4.2.2). A list of the packages we used is available in Data S3.

The PRISE-M questionnaire includes two gender-specific items (“irregular periods” and “erectile dysfunction”). Thus, we first divided the initial sample into male and female groups. We removed the sex-specific item that was missing in each sample from the analysis: “irregular periods” for male participants and “erectile dysfunction” for female participants. First, we computed the raw Spearman correlation coefficients between the total scores of the MADRS, PRISE-M, and YMRS for males and females. Network analyses were conducted in each sample with 31 items of the PRISE-M questionnaire and all MADRS and YMRS items, for a total of 52 items. A power analysis indicated that a sample size of 634 would provide more than 50% statistical power with a 0.8 probability of estimating a network of 52 nodes with a density of 0.08 and edge weights ranging from 0 to 0.3.

A network analysis simultaneously assesses all associations, called edges, between nodes (i.e., the 52 items), without any a priori hypotheses on the associations. We constructed a mixed graph model with the 52 items using Poisson regressions, EBIC model selection, and a hyperparameter *γ* = 0.25, which, unlike other methods, can be applied to discrete variables [39]. In our networks, each node thus represents a symptom, the edges indicate a significant association, and the edge weight represents the signed Poisson regression weight based on standardized data [40]. A graph representation of the network was built using an edge width of 1.4, a repulsion of 0.97, and a maximum edge weight of 1; the same layout was used for both the male and female networks.

We measured the following centrality estimates and reported node centrality raw scores to identify highly interconnected symptoms that may carry a high relative importance in the network.

– Node strength equals the sum of absolute partial correlation coefficients between a node and its neighbor nodes [17]. Node strength is positive, with a higher node strength indicating that the node is more directly connected to other nodes in the network, regardless of the direction of the association.
– Node closeness represents the inverse of the number of edges in the shortest paths (i.e., the minimum number of edges) between one node and all the other nodes [17]. Node closeness is positive, with higher closeness indicating that the node is closer to all the other nodes.
– Node betweenness is the number of shortest paths between two nodes that go through the node in question [17]. Node betweenness is positive, and higher node betweenness indicates that the node contributes more to connecting the other nodes.

In addition to the centrality estimates, we also computed predictability, which is an absolute measure of the variance of a node that can be explained by its neighbor nodes, assuming that all edges go toward this node. Predictability represents the upper bound of shared variance. As a measure of predictability, we report the proportion of explained variance (*R*^2^): 0 means the node is not explained at all by its neighbor nodes, whereas 1 means the variance of the node is completely explained by its neighbor nodes [22]. Predictability can be considered high if *R*^2^ > 0.5, moderate if 0.15 < *R*^2^ ≤ 0.5, and low otherwise [23].

Finally, we assessed the topological overlap [16] to evaluate whether each node measured a construct distinct from those of the rest of the nodes (for more information, see Data S4).

#### 2.3.2. Stability of Network Accuracy and Centrality

Several tools have recently emerged to describe the robustness of a network estimation [41, 42]. They assess two parameters of the network: accuracy and stability of the centrality.

Network accuracy refers to the variability of edge weights within the sample. The confidence intervals of edge weights are measured by bootstrapping to identify edges with consistent and stable weights. We assessed the accuracy of the edge weight estimates by nonparametric bootstrapping (i.e., with replacement) to create 1000 samples. Those samples were used to estimate the 95% confidence intervals and assess the significance of edge weights in our network.

Then, we assessed the stability of the centrality values by case-dropping subset bootstrapping of the samples (i.e., randomly excluding 5%–75% of the sample, *n* = 2500). Stability was measured by the correlation stability coefficient (CS-coefficient), indicating how much data can be removed while maintaining *a* > 0.7 correlation between the original centrality index of the sample and that of the bootstrapped samples with 95% probability [42]. A CS-coefficient > 0.5 indicates high stability of the centrality indices. Results of the network stability analyses are reported as recommended by Burger [40].

#### 2.3.3. Network Clustering and Bridge Centrality

We used network clustering to describe the organization of the items, regardless of the questionnaire they belong to, and identify bridge and stabilizing nodes. We ran a community-detection algorithm called the smart local moving (SLM) algorithm [43], which performs well on small networks (<100 nodes) with only one iteration. We report the conductance, modularity, and coverage of the clusters detected by the SLM method for information, as suggested by previous studies [44]. Conductance measures how intercluster nodes connect, modularity gauges cluster quality, and coverage assesses overall cluster density (Data S5). A lower conductance value suggests better performance of the clustering [44].

We measured node bridge strength, which is similar to node strength but considers only intercluster edges [45]. The simulations of Jones, Ma, and McNally [45] showed that bridge strength can be used to identify bridge nodes, that is, items increasing the likelihood of contagion spreading from one cluster to another [14]. They recommended classifying the five nodes with the highest bridge strength as bridge nodes. Conversely, stabilizing nodes are those that favor the development of their respective cluster. The stabilizing node of each cluster is the node with the largest stabilizing index (i.e., the sum of absolute edge weight values within a cluster [24]). We report the raw scores of the bridge strength and stabilizing index.

## 3. Results

### 3.1. Description of the Sample

**Figure 1:**
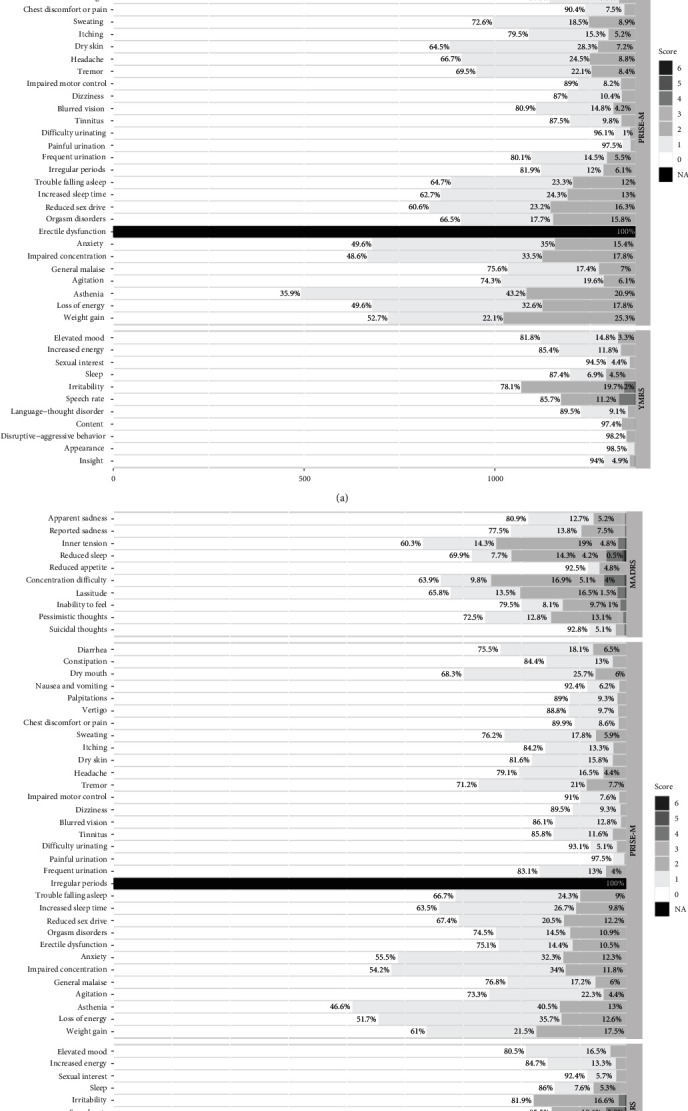
Distribution of the scores in each item of the three questionnaires for males and females. (a) Females (*n* = 1369) and (b) males (*n* = 880).

From among the 4853 initial patients, we excluded 317 with missing data for the MADRS, 10 with missing data for the YMRS, and 377 with missing data for the PRISE-M questionnaires. Finally, we excluded 1900 patients who were not strictly euthymic. Overall, 2249 euthymic outpatients with BD were included. The sample was further split into two subsamples of 880 males and 1369 females described in Table 1. Males and females were very similar in terms of age, MADRS and YMRS total scores, CGI score, medical background, and treatments, ensuring the comparability of the networks. The total score of the MADRS varied mostly between 1 (Q1) and 7 (Q3) and that of the YMRS mostly between 0 (Q1) and 3 (Q3). The residual mood symptoms that were reported the most were inner tension in the MADRS and irritability (males) and elevated mood (females) in the YMRS (Figure 1). The side effects most often reported in the PRISE-M were sedative side effects (asthenia, loss of energy, and impaired concentration).

### 3.2. Description of the Networks

The raw Spearman correlation coefficients were low but positive between the total scores of the YMRS and PRISE-M (0.14 for females and 0.15 for males, *p* < 0.001), and they were moderately positive, that is, 0.3–0.5, between the total scores of the MADRS and PRISE-M (0.44 for females and 0.35 for males, *p* < 0.001; Table S1).

**Figure 2:**
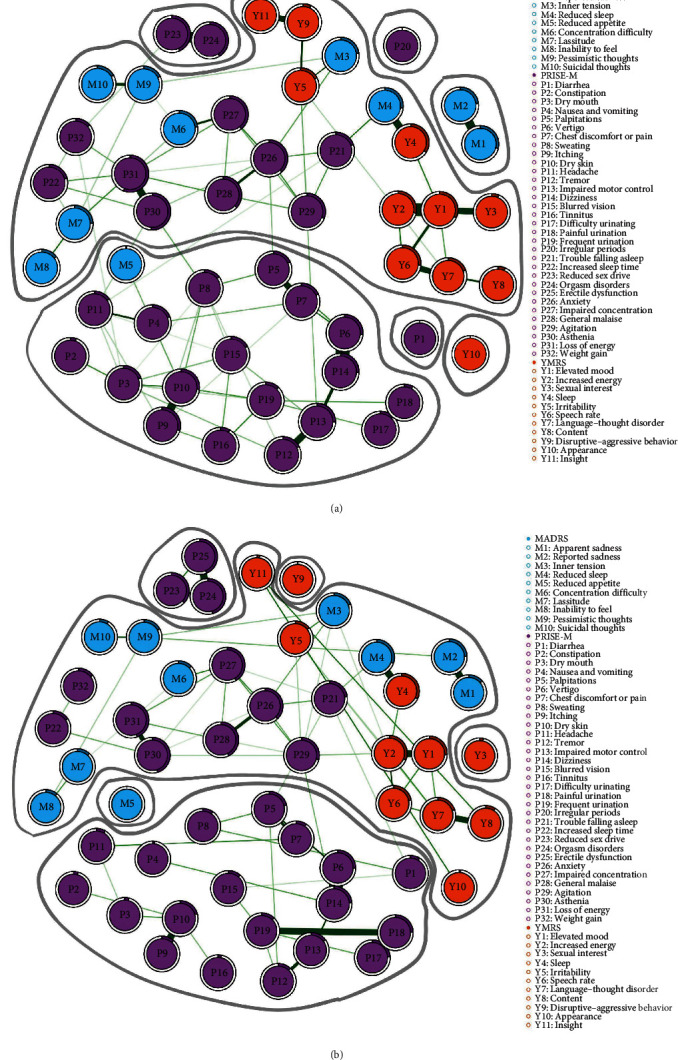
Graphical model of the male and female networks of 52 items of the MADRS, YMRS, and PRISE-M. (a) Females and (b) males. All represented edges have positive weights. Edge width is proportional to edge weight. Of note, a graph is a 2D representation of the network; the relative positions of the nodes and the edge length are arbitrary values to make the graph readable. The colors of the nodes correspond to the three original questionnaires. Surrounded areas correspond to the clusters reported by the community-detection algorithm. Circles around the nodes represent the predictability, available in Table S3.

We compared the connectivity of two networks by computing their density, that is, the ratio of detected edges to the total number of possible edges. The male network showed 5% density, with 71/1326 connections, while the female network showed 6% density, with 85/1326 connections. The two networks had only positive edge weights, with a mean weight of 0.02 (the two adjacency matrices are available in Table S2); the associations between residual depressive and manic symptoms and patient-reported side effects in the network were positive but low. In the male network, the edge weights of 22 of 71 (31%) connections were significant (Data S6). In the female network, 29 of 85 (34%) connections were significant. Graphical representations of the two networks are presented in Figure 2. In addition, in the male network, the topological overlap of the pair of nodes “Painful urination”–”Difficulty urinating” was equal to 0.98 (very close to 1). Therefore, it was more relevant to consider them as measuring the same construct (Table S3). The male and female networks showed broadly the same organization.

**Figure 3:**
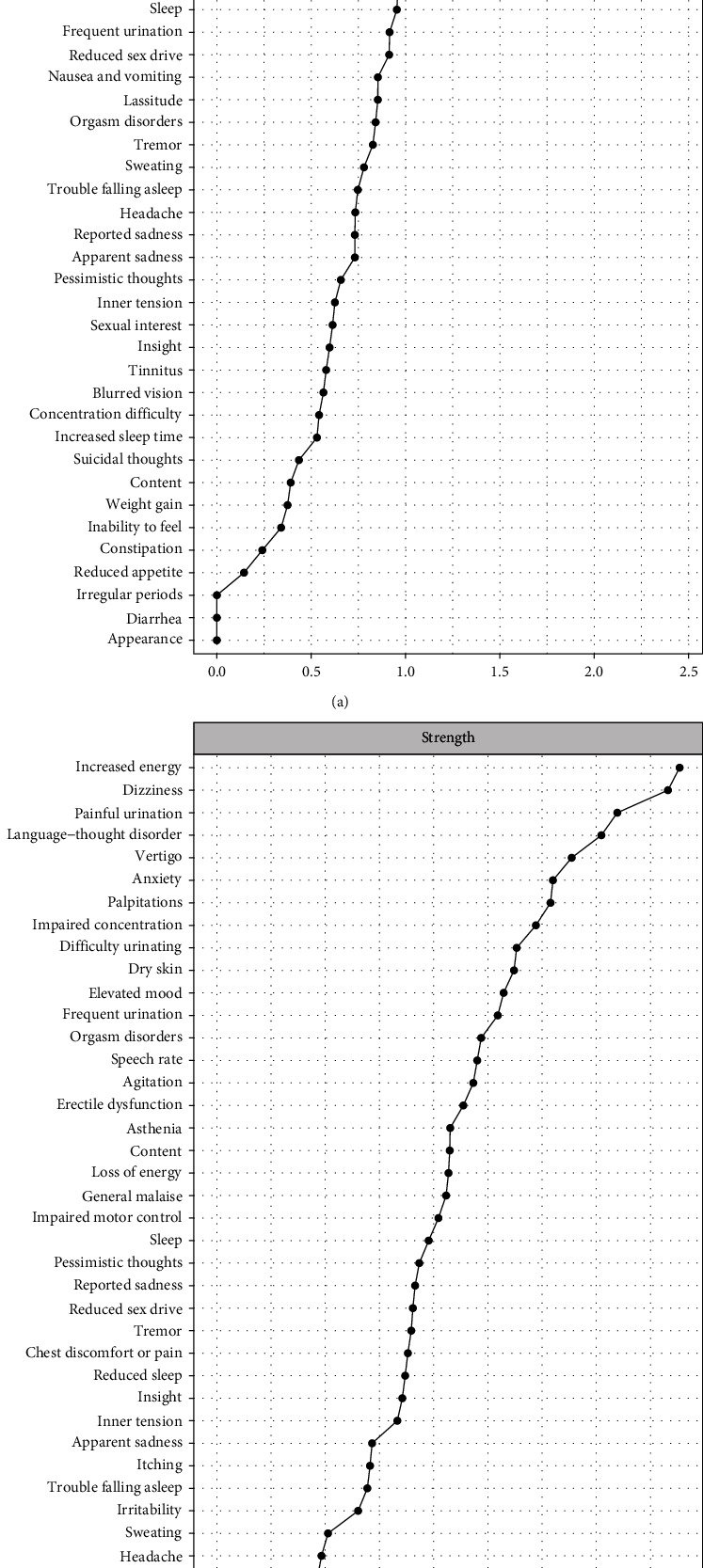
Strength of each node of the network. (a) Females and (b) males. Each line corresponds to one item of the YMRS, MADRS, or PRISE-M questionnaire. Strength is reported as raw scores. Centrality indices were obtained using the *centrality plot* function of the package *bootnet* (Supporting Information).

We then measured the centrality indices (strength, closeness, and betweenness) of each node to identify the most interconnected items and assessed their stability. For the two networks, the CS-coefficients of node betweennesses and node closeness were equal to 0, making the order of node betweenness and closeness impossible to interpret. This suggests that the betweenness and closeness were uniform across all nodes [42] (Figure S1). However, the CS-coefficient of node strengths reached 0.361 in the male network and 0.516 in the female network (Figure S2). Therefore, the order of node strengths was moderately stable in the male network and highly stable in the female network. The strongest node was “increased energy” (YMRS) in the male network and “elevated mood” (YMRS) in the female network (Figure 3). In both networks, the two items were connected to each other, as well as to speech rate (YMRS), suggesting that increased energy and elevated mood are highly interconnected and hold great relative importance in their respective networks. Pairwise comparisons between the strength of each node in the two networks are presented in Figure S3. Finally, we measured predictability as an absolute measure of the variance of the node explained by its neighbor nodes. In the male network, the variance of 26 nodes could be moderately explained by their neighbor nodes, whereas the variance of the remaining 26 nodes was poorly explained by their neighbor nodes (Table S4). In the female network, the variance of 34 nodes was moderately explained by the network, whereas the variance of 16 nodes was poorly explained by the network. In both networks, the items with the highest predictability assessed sedative side effects (asthenia and loss of energy) and sexual functioning (orgasm disorders, erectile dysfunction, and reduced sex drive). Apparent sadness, dry mouth, itching, nausea, and vomiting were moderately more predictable in females, whereas painful urination was moderately more predictable in males. The predictability of the remaining nodes was only slightly or no different between males and females. Overall, the female network showed greater density, higher stability in edge weights and centrality estimates, and more predictable nodes, making inference more robust than for the male network.

### 3.3. Clustering the Networks

Network clustering showed a conductance of 0.04 in the male network and 0.12 in the female network. In both networks, the SLM method provided clusters with a lower conductance than the three clusters formed by the PRISE-M, MADRS, and YMRS questionnaires (Data S7), suggesting that the data-driven clusters better fitted the network than the three original clusters [44]. In the male network, we identified six clusters using the clustering algorithm (Figure 2). We identified a first cluster that included most MADRS and YMRS items and the PRISE-M items that assess sleep, sedation, and restlessness. We named it the “mood” cluster. In addition, 19 PRISE-M items that assess skin, digestive, sensory, urogenital, motor, and cardiovascular side effects formed a second cluster. We named this cluster “nonsexual somatic side effects”. A third cluster was isolated from the other clusters and included the three PRISE-M items that assess sexual functioning, named “sexual side effects”. Finally, three other clusters were constituted by only one isolated item: sexual interest (YMRS), disruptive–aggressive behavior (YMRS), and reduced appetite (MADRS). In the female sample, we identified seven clusters (Figure 2). Similar to the male sample, we identified a “mood” cluster that included most MADRS and YMRS items, along with PRISE-M items related to sleep, sedation, and restlessness, and a nonsexual side effects cluster that included the PRISE-M items that evaluate skin, digestive, sensory, urogenital, motor, and cardiovascular side effects, which additionally included the MADRS item “reduced appetite”. However, reduced appetite (MADRS) showed very low predictability, making its position in the female network unreliable. As in males, a third cluster of the PRISE-M items that assess “sexual side effects” was identified. Three one-node clusters (YMRS “Appearance”, PRISE-M “Irregular periods”, and PRISE-M “diarrhea”) were also isolated. Finally, in contrast to males, “apparent sadness” and “reported sadness” (MADRS) formed a seventh cluster.

We then measured bridge centrality to identify the cluster bridge nodes, that is, the five nodes with the highest bridge strength [45]. By definition, isolated clusters had no bridge nodes. In males, diarrhea, palpitations, dizziness, agitation, and trouble falling asleep (PRISE-M items) represented the only nodes with nonzero bridge strength (Figure S4) and were thus identified as bridge nodes. In females, eight nodes (PRISE-M items) qualified as bridge nodes: asthenia, sweating, headache, weight gain, agitation, impaired motor control, anxiety, and palpitations. We selected eight nodes because agitation, impaired motor control, anxiety, and palpitations showed identical bridge strength values. Palpitations and agitation were bridge nodes for both sexes, serving as links between the “mood” and “nonsexual side effects” clusters.

Finally, in males, increased energy (mood cluster), dizziness (nonsexual side effects cluster), and orgasm disorders (sexual side effects cluster) had the highest stabilizing index within their respective clusters (Table S5), categorizing them as stabilizing nodes. In females, elevated mood (mood cluster), vertigo and dry skin (nonsexual side effects cluster), and orgasm disorders (sexual side effects cluster) had the highest stabilizing index within their respective clusters, identifying them as stabilizing nodes.

An additional analysis was conducted with more conservative euthymia thresholds [28] regarding the MADRS and the YMRS (Data S8). The results remain broadly similar, except for some minor modifications of the clusters' boundaries.

## 4. Discussion

Our primary goal was to gather valuable insights into the relationships between residual mood symptoms and self-reported side effects for males and females with BD during the euthymic phase by conducting a network analysis.

As in previous studies, residual mood symptoms were positively associated with a higher incidence of side effects [46]. The two networks showed poor density relative to that of a previously reported network of mood signs assessed by the YMRS and Quick Inventory of Depressive Symptomatology in the euthymic phase, which showed 18.6% density [20]. This suggests that adding self-reported side effects reduces the global connectivity of the network.

The networks obtained for males and females were highly similar, thus suggesting good reproducibility of the network analyses and reliable results. In addition, the stability and accuracy of our networks suggest that certain significant associations and node strength are likely to be stable across samples. The central items of the male and female networks were increased energy (YMRS) and elevated mood (YMRS), respectively, indicating that in BD, the center of such a network is located in the manic polarity of the residual symptoms. In previously published networks, increased energy and elevated mood showed average-to-high strengths, but were not the strongest nodes [20]. However, they are quite common in the euthymic phase of BD, as the prevalence of increased energy is 11%–19% and that of elevated mood is 8%–33% [2]. Residual manic symptoms are associated with more severe depressive symptoms [47], and more specifically, elevated mood and increased energy are associated with an increased risk of relapse [48]. Our findings suggest a critical role of increased energy and elevated mood in the persistence of other residual mood symptoms and self-reported side effects during the euthymic phase of BD. Prioritizing the treatment of residual manic symptoms in clinical settings could help mitigate other symptoms and complaints.

The differences found between males and females' central items expand the existing knowledge regarding sex differences in the symptomatology of BD; previous studies reported that increased psychomotor activity was greater in men than in women during mania [49]. In females, elevated mood was only associated with other YMRS items, suggesting that subthreshold manic symptoms in females primarily contribute to the development of other manic symptoms. By contrast, in males, increased energy was associated with “agitation” of the PRISE-M and “inner tension” of the MADRS. Agitation is an overlapping symptom of mania and depression and a core feature of mixed mood states, despite its exclusion from the DSM-5 mixed features specifier [50]. Inner tension has also been associated with certain forms of mixed mood states, like Kraeplin and Weygandt's inhibited mania [50] and mixed depressive episodes [51]. Our results suggest that in males, increased energy contributes to the development of mixed residual symptoms, some of which, such as internal tension, are explained by patients as a result of their psychotropic medication [50]. Our results support monitoring of increased energy, especially in males with BD, to prevent the development of additional mixed symptoms and complaints about medication.

In addition, we used predictability as an absolute measure of the quality of the network prediction of one node by all its neighbor nodes. High predictability suggests that a clinical intervention on one node via its neighbors would likely be effective [23]. Our networks can help design interventions towards the reduction of sedation and the enhancement of sexual functioning. More precisely, managing sleep time and anxiety to mitigate asthenia or addressing reduced sex drive to alleviate orgasm disorders may show promise for both males and females, given the moderately high predictability of the nodes. However, 16–26 nodes showed poor predictability, suggesting that their neighbors poorly explain their variability. Thus, further analysis is required to identify what contributes to their variation. In addition, the analysis of topological overlap indicated potential similarities between certain items of the PRISE-M, namely, concerning the constructs of painful urination and difficulty in urinating. Of note, there has yet to be a consensus concerning the threshold for topological overlap, making interpretation challenging [13]. The topological overlap values call for the reconfiguration or redefinition of certain items of the PRISE-M.

We identified similar clusters that fit the male and female networks, suggesting good reproducibility of the community-detection algorithm used in this study. In both networks, we identified a main cluster that included most YMRS items, some MADRS items, and a few PRISE-M items that assess sedation, sleep, and restlessness. These PRISE-M items may be more closely related to the core of residual mood symptoms of BD rather than self-reported medication side effects. The formation of such a cluster can be explained by the fact that patients might incorrectly attribute loss of energy, trouble falling asleep, and impaired concentration to side effects of medication instead of their residual mood symptoms. Therefore, these results suggest that clinicians should consider loss of energy, difficulty falling asleep, and restlessness as mood symptoms rather than side effects, especially when no new medication has been introduced or existing doses have not recently increased. Psychoeducation programs, which usually include several sessions about medication, should also stress this information.

We also identified a second main cluster consisting of nonsexual somatic side effects, suggesting that clinicians should consider skin, digestive, sensory, urogenital, motor, and cardiovascular complaints as having more to do with the side effects of medication than with residual depressive complaints. For both sexes, the two (or three) items of sexual functioning formed a third small cluster, suggesting these complaints should not be considered as residual mood symptoms, but rather authentic side effects that correlate with mood signs in females. In addition, this cluster neither included nor was linked with sexual interest (YMRS), suggesting that overt disinhibited sexual behavior is not to be confused with self-reported satisfying sexual function.

Finally, we identified bridge nodes that connect the three main clusters and provide a better understanding of the interconnections between mood and side effects. For both males and females, palpitations constitute a pathway through which nonsexual side effects are likely to influence residual mood symptoms. Palpitations were linked to the mood cluster through anxiety (females) or trouble falling asleep (males). Symptoms of anxiety frequently co-occur during a manic or depressive episode, especially in females [49] and are a predictor of poor outcome in BD, including greater severity of manic symptoms [52], a longer time to remission [52, 53], more reported side effects of medication [53], more persistent depressive symptoms [54] and increased suicidal ideation [55]. For males, fewer nodes were identified as bridge nodes between mood and nonsexual side effects compared to females. This suggests that the mood of males is less likely to induce more complaints of nonsexual side effects than that of females. Our findings support a potential role of palpitations (along with diarrhea and dizziness in males, and sweating, impaired motor control, and headache in females) in the propagation of side effects to residual mood symptoms. These signs should be particularly monitored by prescribers. We noted that the bridge nodes include side effects that may be induced by serotonin syndrome (including sweating, diarrhea, agitation, etc.). The differences found between males and females in the identification of bridge items may be a consequence of a lack of reproducibility of the bridge node selection method, rather than actual sex-based differences. Further analyses are required to determine whether these side effects genuinely favor the maintenance of residual mood symptoms in BD.

Conversely, agitation, along with trouble falling asleep (males), asthenia, and anxiety (females), are the items for which activation is most likely to lead to increased somatic complaints. They may be considered by prescribers as signs of individual sensitivity to developing and reporting subjective side effects. The reported association between trouble falling asleep and diarrhea in males is noteworthy, as sleep deprivation has been shown to be associated with digestive complications in the general population [56]. In the female network, weight gain could be responsible for the propagation from residual mood symptoms to the sexual side effects cluster through a reduced sex drive. High BMI is associated with impaired sexual function and sexual inactivity in the general population [57]. However, due to the limited number of items (2) that assess sexual functioning in females, further analyses are necessary to better understand how mood symptoms and other side effects affect sexual functioning in BD. Finally, identifying stabilizing nodes revealed many similarities between males and females. Stabilizing nodes, such as vertigo (females) or dizziness (males), could suggest clinical interventions to specifically and efficiently reduce the intensity of a single cluster, such as nonsexual side effects.

Our study had several limitations. The main limitation stems from thresholding the MADRS and YMRS total scores for euthymia, which reduced the variability of mood-related items and degraded the internal consistency of these scales. Other scales may be more suitable for assessing residual symptoms [28], and using them after thresholding the YMRS and MADRS may yield more accurate results. In addition, we used a subjective scale to assess side effects. Future studies could consider including objective scales to assess side effects, such as the UKU side effect rating scale [58], to prevent the contamination of responses by mood state. Finally, we did not assess symptoms of anxiety (apart from one symptom of the PRISE-M named “anxiety”), which represents a potential gap in the assessment of mood symptoms and somatic complaints. The exclusion of treatments from the network is another limitation that may affect the external validity of the results. Certain treatments, such as antidepressants, may account for the observed associations between specific side effects and mood symptoms. Incorporating the classes of treatment did not significantly alter the network. However, the lack of effect is likely due to a lack of statistical power, given the categorical nature of treatment classes and the small number of patients taking each combination of classes. In the present study, we prioritized the investigation of the association between mood symptoms and the PRISE-M. We recommend future studies to investigate the association between mood symptoms and self-reported side effects in patients using a single treatment or class of treatment. Another potential limitation is that the male sample size was smaller than the female sample size, making the comparison of the two networks more difficult [42]. However, the two subsamples were relatively similar, except that more males had type I BD and had experienced psychotic episodes than females, which could have led to differences in the network analyses. Pooling males and females when assessing side effects is one perspective of the study. Another limitation stemmed from predictability, which assumes that (1) all edges are directed toward the node and (2) items do not measure the same underlying construct [22]. We were unable to verify the first requirement because our network was undirected. Therefore, predictability was probably overestimated. Responding to the second requirement, we measured topological overlap to identify overlapping variables [59]. However, there are no guidelines to interpret topological overlap. Future research should focus on bringing clear recommendations on a threshold for topological overlap to guide decisions about node inclusion in the network. Moreover, the reliability of bridge nodes has not been well characterized. Although the cores of the clusters seem reproducible, their boundaries are difficult to establish with community-detection algorithms, especially in case of cluster overlap [46]. Therefore, further research is needed to assess the reliability of bridge nodes. Testing the direction of the associations with longitudinal data to challenge the hypotheses evoked here could be an interesting perspective. Indeed, methodological concerns also arise from using cross-sectional data and emphasize the need for longitudinal studies to establish the directionality of associations between mood and somatic symptoms.

## Figures and Tables

**Table 1:** Description of the sample (*n* = 2249).

Category	Male (*n* = 880)			Female (*n* = 1369)		
	*n* (%)	Mean (SD)	Missing data (%)	*n* (%)	Mean (SD)	Missing data (%)
Age (mean, ±SD)	—	39.1 (12.4)	0	—	39 (12)	0
MADRS (mean, ±SD)	—	4.1 (3.2)	0	—	4.2 (3.2)	0
YMRS (mean, ±SD)	—	1.9 (2.8)	0	—	1.8 (2.7)	0
CGI (mean, ±SD)	—	2.6 (1.4)	4.7	—	2.7 (1.4)	7.4
Type I BD, *n* (%)	477 (54%)	—	0	682 (50%)	—	0
Type II BD, *n* (%)	319 (36%)	—	0	560 (41%)	—	0
Unspecified BD, *n* (%)	84 (10%)	—	0	127 (9%)	—	0
End of the last characterized mood episode >3 months before, *n* (%)	634 (84%)	—	13.8	1006 (85%)	—	13.3
Age at the first mood episode (mean, ±SD)	—	24.4 (9.4)	5.8	—	23.5 (8.8)	8.6
Number of depressive episodes (mean, ±SD)	—	4.9 (5.4)	14.7	—	5.4 (5.5)	19.1
Number of manic episodes (mean, ±SD)	—	1.6 (2.5)	9.4	—	1.1 (2)	12.8
Number of hypomanic episodes (mean, ±SD)	—	3.3 (5.3)	24.5	—	3.3 (5.6)	29.5
Number of mixed episodes (mean, ±SD)	—	0.3 (1.4)	19.2	—	0.4 (1.1)	20.1
Patients with history of psychosis, *n* (%)	352 (47%)	—	15.2	483 (42%)	—	15.7
Number of patients using antidepressants, *n* (%)	204 (28%)	—	18	365 (33%)	—	18.8
Number of patients using mood stabilizers, *n* (%)	336 (47%)	—	18.2	489 (44%)	—	18.6
Number of patients using lithium, *n* (%)	227 (31%)	—	17.8	350 (31%)	—	18.7
Number of patients using antipsychotic, *n* (%)	278 (39%)	—	18	460 (41%)	—	18.8
Number of patients using anxiolytic, *n* (%)	137 (19%)	—	18	246 (22%)	—	18.6
Number of patients using antiparkinsonian drug, *n* (%)	11 (2%)	—	17.4	16 (1%)	—	18.3
Anticholinergic burden (mean, ±SD)	—	1.5 (1.2)	17.4	—	1.5 (1.3)	18.3
Chlorpromazine equivalent, mg/24h (mean, ±SD)	—	103.5 (193.7)	19.7	—	95.2 (218.4)	22.1

Abbreviations: CGI, Clinical Global Impressions scale; MADRS, Montgomery–Asberg Depression Rating scale; YMRS, Young Mania Rating scale.

## Data Availability

Due to ethical and legal restrictions, data involving clinical participants cannot be made publicly available. All relevant data are available upon request to the Fondation FondaMental for researchers who meet the criteria for access to confidential data.

## Supporting Information

Supporting Information Data S1: completed STROBE checklist. Data S2: selection of the anticholinergic burden scale. Data S3: list of the R packages used. Data S4: topological overlap. Data S5: network clustering and conductance of the clusters. Data S6: list of the pair of nodes with significant edge-weights in the male and female networks. Data S7: results of the clustering methods. Data S8: results of the network analysis in male and female samples selected with more conservative thresholds for the MADRS and YMRS (MADRS ≤ 7 and YMRS ≤ 7). Table S1: Spearman correlation coefficients between total scores of each questionnaire in males and females. Table S2: adjacency matrices of the two networks. Table S3: topological overlap values for each pair of nodes. Table S4: predictability of each node in the two networks. Table S5: stabilizing index of each node in the two estimated networks. Figure S1: betweenness and closeness of each node of the network. Figure S2: CS-coefficients for each proportion of the original sample excluded from the case-dropping bootstrap samples (2500 iterations). Figure S3: bootstrapped difference tests (*α* = 0.05) between values of node strength for each pair of nodes. Figure S4: bridge strength of each node in the two networks.
