# Supplementary material for: Exploring the Association Between Residual Mood Symptoms and Self-Reported Side Effects in the Euthymic Phase of Bipolar Disorders: A Cross-Sectional Network Analysis
Source: Depress Anxiety. 2024 Oct 24;2024:3375145. doi: 10.1155/2024/3375145 (PMC11918507; doi:10.1155/2024/3375145)
Supplement: Supporting Information — Data S1: completed STROBE checklist. Data S2: selection of the anticholinergic burden scale. Data S3: list of the R packages used. Data S4: topological overlap. Data S5: network clustering and conductance of the clusters. Data S6: list of the pair of nodes with significant edge-weights in the male and female networks. Data S7: results of the clustering methods. Data S8: results of the network analysis in male and female samples selected with more conservative thresholds for the MADRS and YMRS (MADRS ≤ 7 and YMRS ≤ 7). Table S1: Spearman correlation coefficients between total scores of each questionnaire in males and females. Table S2: adjacency matrices of the two networks. Table S3: topological overlap values for each pair of nodes. Table S4: predictability of each node in the two networks. Table S5: stabilizing index of each node in the two estimated networks. Figure S1: betweenness and closeness of each node of the network. Figure S2: CS-coefficients for each proportion of the original sample excluded from the case-dropping bootstrap samples (2500 iterations). Figure S3: bootstrapped difference tests (α = 0.05) between values of node strength for each pair of nodes. Figure S4: bridge strength of each node in the two networks. [file 3375145.f1.pdf]

Exploring the association between residual mood symptoms and self-reported side effects in the euthymic phase of bipolar disorders: a cross-sectional network analysis

Supplementary information

Supplementary Material SM1: Completed STROBE checklist.

STROBE Statement—checklist of items that should be included in reports of observational studies

	Item No.	Recommendation	Page No.	Relevant text from manuscript
**Title and abstract**	1	(*a*) Indicate the study's design with a commonly used term in the title or the abstract	1	"a cross-sectional network analysis"
		(*b*) Provide in the abstract an informative and balanced summary of what was done and what was found	5	
**Introduction**				
Background/rationale	2	Explain the scientific background and rationale for the investigation being reported	6	first paragraph
Objectives	3	State specific objectives, including any prespecified hypotheses	7	third paragraph
**Methods**				
Study design	4	Present key elements of study design early in the paper	6	"We applied network analysis to side effects and residual depressive and manic symptoms."
Setting	5	Describe the setting, locations, and relevant dates, including periods of recruitment, exposure, follow-up, and data collection	7-9	
Participants	6	(*a*) *Cohort study*—Give the eligibility criteria, and the sources and methods of selection of participants. Describe methods of follow-up<br>*Case-control study*—Give the eligibility criteria, and the sources and methods of case ascertainment and control selection. Give the rationale for the choice of cases and controls<br>*Cross-sectional study*—Give the eligibility criteria, and the sources and methods of selection of participants	8	"Outpatients between 18 and 65 years of age with type I, II, or not otherwise specified (NOS) BD were eligible for the present study. We excluded patients with missing data for the MADRS, the YMRS, or PRISE-M questionnaires. In addition, we excluded non-euthymic patients"
		(*b*) *Cohort study*—For matched studies, give matching criteria and number of exposed and unexposed<br>*Case-control study*—For matched studies, give matching criteria and the number of controls per case		
Variables	7	Clearly define all outcomes, exposures, predictors, potential confounders, and effect modifiers. Give diagnostic criteria, if applicable	8-9	"The diagnosis of BD was based on the Structured Clinical Interview for DSM-IV-TR (SCID) criteria [32]."
Data sources/ measurement	8*	For each variable of interest, give sources of data and details of methods of assessment (measurement). Describe comparability of assessment methods if there is more than one group	8-9	
Bias	9	Describe any efforts to address potential sources of bias	8	"We recorded the sex of the patient (self-report), their age, their type of BD, the total number of mood episodes, the age at onset of BD..."
Study size	10	Explain how the study size was arrived at	10	"A power analysis indicated that a sample size of 634 would provide more than 50% statistical power..."

Continued on next page

Quantitative variables	11	Explain how quantitative variables were handled in the analyses. If applicable, describe which groupings were chosen and why	10	"We constructed a Mixed Graph Model with the 52 items using Poisson regressions, EBIC model selection, and a hyperparameter gamma = 0.25, which, unlike other methods, can be applied to discrete variables [44]."
Statistical methods	12	(*a*) Describe all statistical methods, including those used to control for confounding	10-12	
		(*b*) Describe any methods used to examine subgroups and interactions	12	
		(*c*) Explain how missing data were addressed	8	"We excluded patients with missing data for the MADRS, the YMRS, or PRISE-M questionnaires."
		(*d*) *Cohort study*—If applicable, explain how loss to follow-up was addressed<br>*Case-control study*—If applicable, explain how matching of cases and controls was addressed<br>*Cross-sectional study*—If applicable, describe analytical methods taking account of sampling strategy	11	"we assessed the stability of the centrality values by case-dropping subset bootstrapping of the samples"
		(*e*) Describe any sensitivity analyses		
**Results**				
Participants	13*	(a) Report numbers of individuals at each stage of study—eg numbers potentially eligible, examined for eligibility, confirmed eligible, included in the study, completing follow-up, and analysed	12	"From among the 4,853 initial patients, we excluded 317 with missing data for the MADRS, 10 with missing data for the YMRS, and 377 with missing data for the PRISE-M questionnaires. Finally, we excluded 1,900 patients who were not strictly euthymic. Overall, 2,249 euthymic outpatients with BD were included. The sample was further split into two subsamples of 880 males and 1,369 females."
		(b) Give reasons for non-participation at each stage	12	
		(c) Consider use of a flow diagram		
Descriptive data	14*	(a) Give characteristics of study participants (eg demographic, clinical, social) and information on exposures and potential confounders	Table 1	
		(b) Indicate number of participants with missing data for each variable of interest	Table 1	
		(c) *Cohort study*—Summarise follow-up time (eg, average and total amount)		
Outcome data	15*	*Cohort study*—Report numbers of outcome events or summary measures over time		
		Case-control study—Report numbers in each exposure category, or summary measures of exposure		
		Cross-sectional study—Report numbers of outcome events or summary measures	Figure 1	
Main results	16	(*a*) Give unadjusted estimates and, if applicable, confounder-adjusted estimates and their precision (eg, 95% confidence interval). Make clear which confounders were adjusted for and why they were included	13-14	
		(*b*) Report category boundaries when continuous variables were categorized		
		(*c*) If relevant, consider translating estimates of relative risk into absolute risk for a meaningful time period		

Continued on next page

Other analyses	17	Report other analyses done—eg analyses of subgroups and interactions, and sensitivity analyses	Supplementary Material	
**Discussion**				
Key results	18	Summarise key results with reference to study objectives	15-16	
Limitations	19	Discuss limitations of the study, taking into account sources of potential bias or imprecision. Discuss both direction and magnitude of any potential bias	18-19	
Interpretation	20	Give a cautious overall interpretation of results considering objectives, limitations, multiplicity of analyses, results from similar studies, and other relevant evidence	16-19	"Our findings suggest a critical role of increased energy and elevated mood in the persistence of other residual mood symptoms and self-reported side effects during the euthymic phase of BD."
Generalisability	21	Discuss the generalisability (external validity) of the study results	19	"The exclusion of treatments from the network is another limitation that may affect the external validity of the results."
**Other information**				
Funding	22	Give the source of funding and the role of the funders for the present study and, if applicable, for the original study on which the present article is based	20	

*Give information separately for cases and controls in case-control studies and, if applicable, for exposed and unexposed groups in cohort and cross-sectional studies.

Note: An Explanation and Elaboration article discusses each checklist item and gives methodological background and published examples of transparent reporting. The STROBE checklist is best used in conjunction with this article (freely available on the Web sites of PLoS Medicine at http://www.plosmedicine.org/, Annals of Internal Medicine at http://www.annals.org/, and Epidemiology at http://www.epidem.com/). Information on the STROBE Initiative is available at www.strobe-statement.org.

Supplementary Material SM2: Selection of the anticholinergic burden scale.

Salahudeen’s scale showed good concurrent validity in assessing anticholinergic side effects in Bipolar Disorders (BD) [1]. As in this previous study, when Salahudeen’s scale assigns multiple scores to one medication, we select the minimum: for example, “1 or 2” was coded as 1 and “2 or 3” was coded as 2. The anticholinergic burden score of a patient was the cumulative score of their medications. Drugs not included in Salahudeen’s scale had an anticholinergic burden score of 0, as if they had no anticholinergic properties according to the scale, like in a previous study [2].

Supplementary Material SM3: List of the R packages used.

- The network power analysis was conducted with the *powerly* function of the *powerly* package [3] (version 1.8.6).
- The MGM network was estimated using the *estimateNetwork* function of the package *bootnet* [4] (version 1.5).
- A graph representation of the network (shown in Fig. 2) was built using the *qgraph* package [5] (version 1.9.5).
- We calculated node centrality raw scores using the *centralityPlot* function of the *qgraph* package [5] (version 1.9.5).
- The predictability of each node was estimated with the *predict.mgm* function of the *mgm* package [6,7] (version 1.2-14).
- We measured node bridge strength with the *bridge* function of the *networktools* R package [8] (version 1.5.1).

Supplementary Material SM4: Topological overlap.

Topological overlap is one method to assess the similarity between two nodes. We used the formula of Zhang & Horvath [9] for weighted network analysis. According to their definition, two nodes are highly topologically overlapped if they are connected with each other and share similar connections with the other nodes in the network. Topological overlap varies from 0 to 1: 0 indicates that the two nodes are not associated and do not share common neighbors, whereas a topological overlap equal to 1 indicates that the two nodes are associated (with an edge weight of 1) and share exactly the same neighbors. Measuring topological overlap is recommended in network analysis because it helps make inferences based on the network. Indeed, if topological overlap of one pair of nodes is close to 1, the edge weight between the two nodes is a measure of similarity and not a measure of

association between two distinct variables [10]. There is no existing threshold for topological overlap, therefore we reported topological overlap for information and discussed it.

Supplementary Material SM5: Network clustering and conductance of the clusters.

We used the Smart Local Moving algorithm or SLM [11], which is a modularity optimization method. SLM is based on the Louvain algorithm [12] completed by the multilevel refinement method [13]. As opposed to the Louvain algorithm, SLM can split the communities defined in a previous iteration for an increase in modularity. Modularity is the fraction of intra-cluster edges minus the expected probability that those edges exist in a random graph and represents the density of the clusters compared to the rest of the network. The SLM method was defined as more performant than the Infomap algorithm in a comparative analysis of clustering algorithms [14]. We ran SLM on Java 1.8.0 with the "*Modularity Optimizer JAR file"* available at http://www.ludowaltman.nl/slm/ (by Waltman & van Eck, update of 2015). We used 10-fold cross validation to optimize the standard modularity function with a standard resolution of 0.1.

To assess the clustering performance, we measured the conductance of the clusters suggested by the SLM method. The conductance of one cluster is the number of inter-cluster edges of one cluster divided by either, the number of edges with an endpoint in the cluster, or the number of edges that do not have an endpoint in the cluster, whichever is smaller. The conductance of a clustering algorithm was the sum of the conductances of each cluster identified. The closer to 0 the conductance is, the more performant the clustering [14]. The conductance of the clusters identified by the SLM was 0.04 (males) and 0.12 (females), which was better than the conductance of the three original communities on both networks (0.36 in males, 0.37 in females, see the results below).

Supplementary Material SM6: List of the pair of nodes with significant edge-weights in the male and female networks.

In the male network:

1. Increased Energy & Speech rate
2. Anxiety & General malaise
3. Anxiety & Impaired concentration
4. Apparent sadness & Reported sadness
5. Asthenia & Loss of energy
6. Concentration difficulty & Impaired concentration

7. Difficulty urinating & Painful urination
8. Elevated Mood & Increased Energy
9. Elevated Mood & Speech rate
10. Impaired concentration & Agitation
11. Impaired concentration & Loss of energy
12. Irritability & Inner tension
13. Itching & Dry skin
14. Lassitude & Inability to feel
15. Orgasm disorders & Erectile dysfunction
16. Pessimistic thoughts & Suicidal thoughts
17. Reduced sex drive & Erectile dysfunction
18. Reduced sex drive & Orgasm disorders
19. Sleep & Reduced sleep
20. Speech rate & Language-Thought Disorder
21. Tremor & Impaired motor control
22. Vertigo & Dizziness

In the female network:

1. Increased Energy & Speech rate
2. Anxiety & General malaise
3. Anxiety & Impaired concentration
4. Apparent sadness & Reported sadness
5. Asthenia & Loss of energy
6. Concentration difficulty & Impaired concentration
7. Constipation & Dry mouth
8. Dry mouth & Dry skin
9. Elevated Mood & Increased Energy
10. Elevated Mood & Sexual Interest
11. Elevated Mood & Speech rate
12. Impaired concentration & General malaise
13. Impaired concentration & Loss of energy
14. Impaired motor control & Dizziness
15. Inner tension & Anxiety
16. Irritability & Inner tension
17. Itching & Dry skin
18. Lassitude & Inability to feel
19. Nausea and vomiting & Headache

20. Palpitations & Chest discomfort or pain
21. Pessimistic thoughts & Suicidal thoughts
22. Reduced sex drive & Orgasm disorders
23. Reduced sleep & Trouble falling asleep
24. Sleep & Reduced sleep
25. Speech rate & Language-Thought Disorder
26. Tremor & Impaired motor control
27. Trouble falling asleep & Agitation
28. Trouble falling asleep & Anxiety
29. Vertigo & Dizziness

Supplementary Material SM7: Results of the clustering methods.

Sample	Clustering method	Number of clusters	Coverage	Modularity	Conductance
Male	Distributing the items in the 3 questionnaires YMRS, MADRS or PRISE-M	3	0.87	0.39	**0.36**
	SLM method	6	0.96	0.45	**0.04**
Female	Distributing the items in the 3 questionnaires YMRS, MADRS or PRISE-M	3	0.89	0.33	**0.37**
	SLM method	7	0.92	0.44	**0.12**

Coverage is the fraction of intra-cluster edges on the total number of edges in the graph and represents the global density of all the clusters.

Supplementary Material SM8: Results of the network analysis in male and female samples selected with more conservative thresholds for the MADRS and YMRS (MADRS ≤ 7 and YMRS ≤ 7).

	**Male network with MADRS ≤ 7 and YMRS ≤ 7 (n = 659)**	**Female network with MADRS ≤ 7 and YMRS ≤ 7 (n = 1016)**
**Graphical representation of the network** (the color of the nodes represent identified clusters)	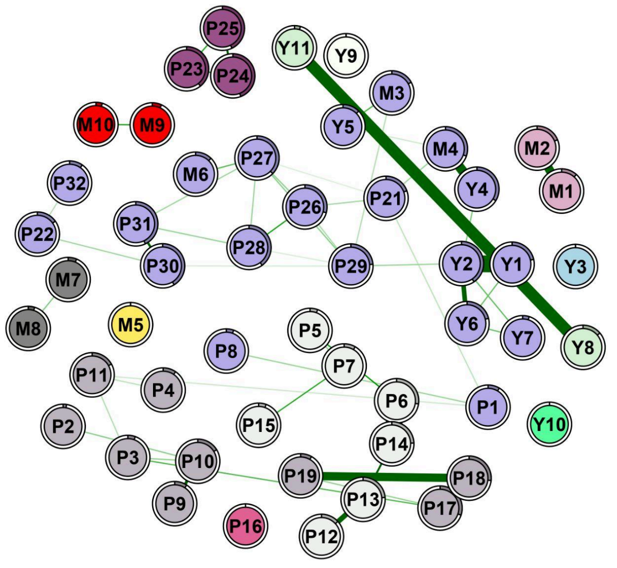	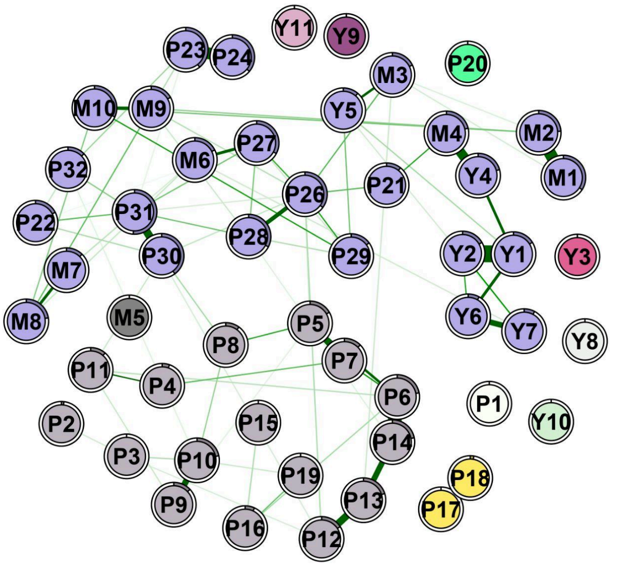
**Top 5 node strength**	1. Increased energy<br>2. Painful urination<br>3. Difficulty urinating<br>4. Dizziness<br>5. Vertigo	1. Elevated mood<br>2. Anxiety<br>3. Vertigo<br>4. Increased energy<br>5. Loss of energy
**Top 5 predictability**	1. Orgasm disorders (R2 = 0.441)<br>2. Erectile dysfunction (R2 = 0.429)<br>3. Loss of energy (R2 = 0.429)<br>4. Asthenia (R2 = 0.413)<br>5. Increased energy (R2 = 0.404)	1. Loss of energy (R2 = 0.510)<br>2. Asthenia (R2 = 0.399)<br>3. Reduced sex drive (R2 = 0.393)<br>4. Orgasm disorders (R2 = 0.361)<br>5. Anxiety (R2 = 0.351)
**Top 5 bridge strength**	1. Diarrhea (0.11)<br>2. Headache (0.11)	1. Asthenia (0.19)<br>2. Anxiety (0.18)<br>3. Tremor (0.16)<br>4. Sweating (0.12)<br>5. Palpitations (0.07)
**Top 5 stabilizing index**	1. Increased energy (2.62)<br>2. Painful urination (1.80)<br>3. Difficulty urinating (1.67)<br>4. Dizziness (1.65)<br>5. Vertigo (1.63)	1. Elevated mood (2.06)<br>2. Vertigo (1.91)<br>3. Increased energy (1.74)<br>4. Anxiety (1.73)<br>5. Loss of energy (1.58)

Note. To identify clusters, we ran an SLM clustering method with the same parameters as in the original analysis.

References

1 Vidal N, Brunet-Gouet E, Frileux S, Aouizerate B, Aubin V, Belzeaux R, et al. Comparative analysis of anticholinergic burden scales to explain iatrogenic cognitive impairment and self-reported side effects in the euthymic phase of bipolar disorders: Results from the FACE-BD cohort. Eur Neuropsychopharmacol. 2023 Dec;77:67–79.
2 Lisibach A, Gallucci G, Beeler PE, Csajka C, Lutters M. High anticholinergic burden at admission associated with in-hospital mortality in older patients: A comparison of 19 different anticholinergic burden scales. Basic Clin Pharmacol Toxicol. 2022 Feb;130(2):288–300.
3 Constantin MA, Schuurman NK, Vermunt JK. A general Monte Carlo method for sample size analysis in the context of network models. Psychol Methods. 2023 Jul DOI: 10.1037/met0000555
4 Epskamp S, Borsboom D, Fried EI. Estimating psychological networks and their accuracy: A tutorial paper. Behav Res Methods. 2018 Feb;50(1):195–212.
5 Epskamp S, Cramer AOJ, Waldorp LJ, Schmittmann VD, Borsboom D. qgraph: Network Visualizations of Relationships in Psychometric Data. J Stat Softw. 2012 May;48(4):1–18.
6 Haslbeck JMB, Waldorp LJ. mgm: Estimating Time-Varying Mixed Graphical Models in High-Dimensional Data. J Stat Softw. 2020 Apr;93(8):1–46.
7 Haslbeck JMB, Waldorp LJ. mgm: structure estimation for time-varying mixed graphical models in high-dimensional data. arXiv:151006871. 2016
8 Jones PJ, Ma R, McNally RJ. Bridge Centrality: A Network Approach to Understanding Comorbidity. Multivar Behav Res. 2021 Apr;56(2):353–67.
9 Zhang B, Horvath S. A general framework for weighted gene co-expression network analysis. Stat Appl Genet Mol Biol. 2005;4:Article17.
10 Fried EI, Cramer AOJ. Moving Forward: Challenges and Directions for Psychopathological Network Theory and Methodology. Perspect Psychol Sci J Assoc Psychol Sci. 2017 Nov;12(6):999–1020.
11 Waltman L, van Eck NJ. A smart local moving algorithm for large-scale modularity-based community detection. Eur Phys J B. 2013 Nov;86(11):471.
12 Blondel VD, Guillaume J-L, Lambiotte R, Lefebvre E. Fast unfolding of communities in large networks. J Stat Mech Theory Exp. 2008 Oct;2008(10):P10008.
13 Rotta R, Noack A. Multilevel Local Search Algorithms for Modularity Clustering. ACM J Exp Algorithmics. 2011 Jul;16. DOI: 10.1145/1963190.1970376
14 Emmons S, Kobourov S, Gallant M, Börner K. Analysis of Network Clustering Algorithms and Cluster Quality Metrics at Scale. PLOS ONE. 2016 Jul;11(7):e0159161.

Supplementary Table 1. Spearman correlation coefficients between total scores of each questionnaire in males and females. The values for females are in the top right corner and the values for males are in the bottom left corner.

Males/females	YMRS	MADRS	PRISE-M
YMRS	-	0.2	0.14
MADRS	0.18	-	0.44
PRISE-M	0.15	0.35	-

Supplementary Table 2. Adjacency matrices of the two networks. The top-right side of the matrix is the female adjacency matrix. The bottom-left corner of the matrix is the male adjacency matrix.

Males \ Females	Elevated Mood	Increased Energy	Sexual Interest	Sleep	Irritability	Speech rate	Language-Thought Disorder	Content	Disruptive-Aggressive Behavior	Appearance	Insight	Apparent sadness	Reported sadness	Inner tension	Reduced sleep	Reduced appetite	Concentration difficulty	Lassitude	Inability to feel	Pessimistic thoughts	Suicidal thoughts	Diarrhea	Constipation	Dry mouth	Nausea and vomiting	Palpitations	Vertigo	Chest discomfort or pain	Sweating	Itching	Dry skin	Headache	Tremor	Impaired motor control	Dizziness	Blurred vision	Tinnitus	Difficulty urinating	Painful urination	Frequent urination	Trouble falling asleep	Increased sleep time	Reduced sex drive	Orgasm disorders	Irregular periods OR Erectile dysfunction	Anxiety	Impaired concentration	General malaise	Agitation	Asthenia	Loss of energy	Weight gain
Elevated Mood	-	0,93	0,61	0,29	0	0,43	0,19	0	0	0	0	0	0	0	0	0	0	0	0	0	0	0	0	0	0	0	0	0	0	0	0	0	0	0	0	0	0	0	0	0	0	0	0	0	0	0	0	0	0	0	0	0
Increased Energy	0,71	-	0	0	0	0,32	0	0	0	0	0	0	0	0	0	0	0	0	0	0	0	0	0	0	0	0	0	0	0	0	0	0	0	0	0	0	0	0	0	0	0	0	0	0	0	0	0	0	0	0	0	0
Sexual Interest	0	0	-	0	0	0	0	0	0	0	0	0	0	0	0	0	0	0	0	0	0	0	0	0	0	0	0	0	0	0	0	0	0	0	0	0	0	0	0	0	0	0	0	0	0	0	0	0	0	0	0	0
Sleep	0	0,27	0	-	0	0	0	0	0	0	0	0	0	0	0,66	0	0	0	0	0	0	0	0	0	0	0	0	0	0	0	0	0	0	0	0	0	0	0	0	0	0	0	0	0	0	0	0	0	0	0	0	0
Irritability	0	0,10	0	0	-	0	0,08	0	0,39	0	0	0	0	0,36	0	0	0	0	0	0	0	0	0	0	0	0	0	0	0	0	0	0	0	0	0	0	0	0	0	0	0	0	0	0	0	0	0	0	0,16	0	0	0
Speech rate	0,35	0,47	0	0	0	-	0,59	0	0	0	0	0	0	0	0	0	0	0	0	0	0	0	0	0	0	0	0	0	0	0	0	0	0	0	0	0	0	0	0	0	0	0	0	0	0	0	0	0	0	0	0	0
Language-Thought Disorder	0,26	0,34	0	0	0,13	0,38	-	0,39	0	0	0	0	0	0	0	0	0	0	0	0	0	0	0	0	0	0	0	0	0	0	0	0	0	0	0	0	0	0	0	0	0	0	0	0	0	0	0	0	0	0	0	0
Content	0	0	0	0	0	0	0,67	-	0	0	0	0	0	0	0	0	0	0	0	0	0	0	0	0	0	0	0	0	0	0	0	0	0	0	0	0	0	0	0	0	0	0	0	0	0	0	0	0	0	0	0	0
Disruptive-Aggressive Behavior	0	0	0	0	0	0	0	0	-	0	0,60	0	0	0	0	0	0	0	0	0	0	0	0	0	0	0	0	0	0	0	0	0	0	0	0	0	0	0	0	0	0	0	0	0	0	0	0	0	0	0	0	0
Appearance	0	0	0	0	0	0	0	0	0	-	0	0	0	0	0	0	0	0	0	0	0	0	0	0	0	0	0	0	0	0	0	0	0	0	0	0	0	0	0	0	0	0	0	0	0	0	0	0	0	0	0	0
Insight	0	0	0	0	0	0	0	0,41	0	0,45	-	0	0	0	0	0	0	0	0	0	0	0	0	0	0	0	0	0	0	0	0	0	0	0	0	0	0	0	0	0	0	0	0	0	0	0	0	0	0	0	0	0
Apparent sadness	0	0	0	0	0	0	0	0	0	0	0	-	0,73	0	0	0	0	0	0	0	0	0	0	0	0	0	0	0	0	0	0	0	0	0	0	0	0	0	0	0	0	0	0	0	0	0	0	0	0	0	0	0
Reported sadness	0	0	0	0	0	0	0	0	0	0	0	0,72	-	0	0	0	0	0	0	0	0	0	0	0	0	0	0	0	0	0	0	0	0	0	0	0	0	0	0	0	0	0	0	0	0	0	0	0	0	0	0	0
Inner tension	0	0,08	0	0	0,31	0	0	0	0	0	0	0	0	-	0	0	0	0	0	0,09	0	0	0	0	0	0	0	0	0	0	0	0	0	0	0	0	0	0	0	0	0	0	0	0	0	0,18	0	0	0	0	0	0
Reduced sleep	0	0	0	0,70	0	0	0	0	0	0	0	0	0	0	-	0	0	0	0	0	0	0	0	0	0	0	0	0	0	0	0	0	0	0	0	0	0	0	0	0	0,30	0	0	0	0	0	0	0	0	0	0	0
Reduced appetite	0	0	0	0	0	0	0	0	0	0	0	0	0	0	0	-	0	0	0	0	0	0	0	0	0,14	0	0	0	0	0	0	0	0	0	0	0	0	0	0	0	0	0	0	0	0	0	0	0	0	0	0	0
Concentration difficulty	0	0	0	0	0	0	0	0	0	0	0	0	0	0	0	0	-	0,10	0	0,04	0	0	0	0	0	0	0	0	0	0	0	0	0	0	0	0	0	0	0	0	0	0	0	0	0	0	0,40	0	0	0	0	0
Lassitude	0	0	0	0	0	0	0	0	0	0	0	0	0	0	0	0	0,06	-	0,34	0,09	0	0	0	0	0	0	0	0	0	0	0	0	0	0	0	0	0	0	0	0	0	0,08	0	0	0	0	0	0	0	0	0,24	0
Inability to feel	0	0	0	0	0	0	0	0	0	0	0	0	0	0	0	0	0	0,28	-	0	0	0	0	0	0	0	0	0	0	0	0	0	0	0	0	0	0	0	0	0	0	0	0	0	0	0	0	0	0	0	0	0
Pessimistic thoughts	0	0	0	0	0	0	0	0	0	0	0	0	0,20	0,15	0	0	0	0	0,12	-	0,44	0	0	0	0	0	0	0	0	0	0	0	0	0	0	0	0	0	0	0	0	0	0	0	0	0	0	0	0	0	0	0
Suicidal thoughts	0	0	0	0	0	0	0	0	0	0	0	0	0	0	0	0	0	0	0	0,46	-	0	0	0	0	0	0	0	0	0	0	0	0	0	0	0	0	0	0	0	0	0	0	0	0	0	0	0	0	0	0	0
Diarrhea	0	0	0	0	0	0	0	0	0	0	0	0	0	0	0	0	0	0	0	0	0	-	0	0	0	0	0	0	0	0	0	0	0	0	0	0	0	0	0	0	0	0	0	0	0	0	0	0	0	0	0	0
Constipation	0	0	0	0	0	0	0	0	0	0	0	0	0	0	0	0	0	0	0	0	0	0	-	0,24	0	0	0	0	0	0	0	0	0	0	0	0	0	0	0	0	0	0	0	0	0	0	0	0	0	0	0	0
Dry mouth	0	0	0	0	0	0	0	0	0	0	0	0	0	0	0	0	0	0	0	0	0	0	0	-	0	0	0	0	0,04	0	0,29	0,07	0,12	0	0	0,14	0	0	0	0,08	0	0	0	0	0	0	0	0	0	0	0	0,06
Nausea and vomiting	0	0	0	0	0	0	0	0	0	0	0	0	0	0	0	0	0	0	0	0	0	0	0	0	-	0	0	0,15	0	0	0,17	0,39	0	0	0	0	0	0	0	0	0	0	0	0	0	0	0	0	0	0	0	0
Palpitations	0	0	0	0	0	0	0	0	0	0	0	0	0	0	0	0	0	0	0	0	0	0	0	0	0	-	0,25	0,60	0,18	0	0	0	0	0	0	0	0	0	0	0	0	0	0	0	0	0,13	0	0	0	0	0	0
Vertigo	0	0	0	0	0	0	0	0	0	0	0	0	0	0	0	0	0	0	0	0	0	0	0	0	0	0,47	-	0,34	0	0	0	0	0	0	0,98	0	0	0	0	0	0	0	0	0	0	0	0	0	0	0	0	0
Chest discomfort or pain	0	0	0	0	0	0	0	0	0	0	0	0	0	0	0	0	0	0	0	0	0	0	0	0	0	0,52	0	-	0	0,06	0	0	0	0	0	0	0	0	0	0	0	0	0	0	0	0	0	0	0	0	0	0
Sweating	0	0	0	0	0	0	0	0	0	0	0	0	0	0	0	0	0	0	0	0	0	0,25	0	0	0	0,26	0	0	-	0,09	0,16	0	0,06	0	0	0,07	0	0	0	0	0	0	0	0	0	0	0	0	0	0,16	0	0
Itching	0	0	0	0	0	0	0	0	0	0	0	0	0	0	0	0	0	0	0	0	0	0	0	0	0	0	0	0	0	-	0,70	0,08	0	0	0	0	0,15	0	0	0	0	0	0	0	0	0	0	0	0	0	0	0
Dry skin	0	0	0	0	0	0	0	0	0	0	0	0	0	0	0	0	0	0	0	0	0	0	0,20	0,28	0	0	0	0	0	0,71	-	0	0	0	0	0	0	0	0	0,23	0	0	0	0	0	0	0	0	0	0	0	0
Headache	0	0	0	0	0	0	0	0	0	0	0	0	0	0	0	0	0	0	0	0	0	0	0	0,12	0	0	0	0,36	0	0	0	-	0	0	0	0,04	0	0	0	0	0,05	0	0	0	0	0	0	0	0	0,09	0	0
Tremor	0	0	0	0	0	0	0	0	0	0	0	0	0	0	0	0	0	0	0	0	0	0	0	0	0	0,18	0	0	0	0	0	0	-	0,64	0	0	0	0	0	0	0	0	0	0	0	0	0	0	0	0	0	0
Impaired motor control	0	0	0	0	0	0	0	0	0	0	0	0	0	0	0	0	0	0	0	0	0	0	0	0	0	0	0	0	0	0	0	0	0,55	-	0,45	0,13	0	0	0	0	0	0	0	0	0	0	0	0	0,13	0	0	0
Dizziness	0	0	0	0	0	0	0	0	0	0	0	0	0	0	0	0	0	0	0	0	0	0	0	0	0,27	0	1,00	0	0	0	0	0	0	0,47	-	0	0	0	0	0	0	0	0	0	0	0	0	0	0	0	0	0
Blurred vision	0	0	0	0	0	0	0	0	0	0	0	0	0	0	0	0	0	0	0	0	0	0,08	0	0	0	0	0	0	0	0	0	0	0	0	0	-	0,18	0	0	0	0	0	0	0	0	0	0	0	0	0	0	0
Tinnitus	0	0	0	0	0	0	0	0	0	0	0	0	0	0	0	0	0	0	0	0	0	0	0	0	0	0	0	0	0	0	0,18	0	0	0	0	0	-	0	0	0,26	0	0	0	0	0	0	0	0	0	0	0	0
Difficulty urinating	0	0	0	0	0	0	0	0	0	0	0	0	0	0	0	0	0	0	0	0	0	0	0	0	0	0	0	0	0	0	0	0	0	0	0	0	0	-	1,00	0,16	0	0	0	0	0	0	0	0	0	0	0	0
Painful urination	0	0	0	0	0	0	0	0	0	0	0	0	0	0	0	0	0	0	0	0	0	0	0	0	0	0	0	0	0	0	0	0	0	0	0	0	0	1,00	-	0,19	0	0	0	0	0	0	0	0	0	0	0	0
Frequent urination	0	0	0	0	0	0	0	0	0	0	0	0	0	0	0	0	0	0	0	0	0	0	0	0	0	0	0	0	0	0	0	0	0,17	0	0	0,20	0	0,23	0,69	-	0	0	0	0	0	0	0	0	0	0	0	0
Trouble falling asleep	0	0	0	0	0	0	0	0	0	0	0	0	0	0	0,17	0	0	0	0	0	0	0,10	0	0	0	0,11	0	0	0	0	0	0	0	0	0	0	0	0	0	0	-	0	0	0	0	0,20	0	0	0,19	0	0	0
Increased sleep time	0	0	0	0	0	0	0	0	0	0	0	0	0	0	0	0	0	0	0	0	0	0	0	0	0	0	0	0	0	0	0	0	0	0	0	0	0	0	0	0	0	-	0	0	0	0	0	0	0	0,17	0,19	0,09
Reduced sex drive	0	0	0	0	0	0	0	0	0	0	0	0	0	0	0	0	0	0	0	0	0	0	0	0	0	0	0	0	0	0	0	0	0	0	0	0	0	0	0	0	0	0	-	0,84	0	0	0	0	0	0	0	0,07
Orgasm disorders	0	0	0	0	0	0	0	0	0	0	0	0	0	0	0	0	0	0	0	0	0	0	0	0	0	0	0	0	0	0	0	0	0	0	0	0	0	0	0	0	0	0	0,49	-	0	0	0	0	0	0	0	0
Erectile Dysfunction OR Irregular periods	0	0	0	0	0	0	0	0	0	0	0	0	0	0	0	0	0	0	0	0	0	0	0	0	0	0	0	0	0	0	0	0	0	0	0	0	0	0	0	0	0	0	0,41	0,73	-	0	0	0	0	0	0	0
Anxiety	0	0	0	0	0	0	0	0	0	0	0	0	0	0,20	0	0	0	0	0	0	0	0	0	0	0	0	0	0	0	0	0	0	0	0	0	0	0	0	0	0	0,24	0	0	0	0	-	0,27	0,42	0,24	0,10	0,06	0
Impaired concentration	0	0	0	0	0	0	0	0	0	0	0	0	0	0	0	0	0,33	0	0	0	0	0	0	0	0	0	0	0	0	0	0	0	0	0	0	0	0	0	0	0	0,07	0	0	0	0	0,30	-	0,16	0,15	0	0,17	0
General malaise	0	0	0	0	0	0	0	0	0	0	0	0	0	0	0	0	0	0	0	0	0	0	0	0	0	0	0	0	0	0	0	0	0	0	0	0	0	0	0	0	0	0	0	0	0	0,54	0,20	-	0,18	0	0,21	0
Agitation	0	0,16	0	0	0,11	0	0	0	0	0	0	0	0	0,08	0	0	0	0	0	0	0	0	0	0	0	0	0	0	0	0	0	0	0	0	0,18	0	0	0	0	0	0	0	0	0	0	0,19	0,25	0,09	-	0	0	0
Asthenia	0	0	0	0	0	0	0	0	0	0	0	0	0	0	0	0	0	0	0	0	0	0	0	0	0	0	0	0	0	0	0	0	0	0	0	0	0	0	0	0	0	0,21	0	0	0	0,06	0,08	0	0,12	-	0,67	0
Loss of energy	0	0	0	0	0	0	0	0	0	0	0	0	0	0	0	0	0	0	0	0	0	0	0	0	0	0	0	0	0	0	0	0	0	0	0	0	0	0	0	0	0	0	0	0	0	0	0,24	0,22	0	0,61	-	0,16
Weight gain	0	0	0	0	0	0	0	0	0	0	0	0	0	0	0	0	0	0	0	0	0	0	0	0	0	0	0	0	0	0	0	0	0	0	0	0	0	0	0	0	0	0,15	0	0	0	0	0	0	0	0	0	-

Supplementary Table 3. Topological overlap values for each pair of nodes.

Females / Males	Elevated Mood	Increased Energy	Sexual Interest	Sleep	Irritability	Speech rate	Language-Thought Disorder	Content	Disruptive-Aggressive Behavior	Appearance	Insight	Apparent sadness	Reported sadness	Inner tension	Reduced sleep	Reduced appetite	Concentration difficulty	Lassitude	Inability to feel	Pessimistic thoughts	Suicidal thoughts	Diarrhea	Constipation	Dry mouth	Nausea and vomiting	Palpitations	Vertigo	Chest discomfort or pain	Sweating	Itching	Dry skin	Headache	Tremor	Impaired motor control	Dizziness	Blurred vision	Tinnitus	Difficulty urinating	Painful urination	Frequent urination	Trouble falling asleep	Increased sleep time	Reduced sex drive	Orgasm disorders	Irregular periods / Erectile dysfunction	Anxiety	Impaired concentration	General malaise	Agitation	Asthenia	Loss of energy	Weight gain
Elevated Mood	-	0,81	0,61	0,18	0,01	0,44	0,21	0,05	0	0	0	0	0	0	0,10	0	0	0	0	0	0	0	0	0	0	0	0	0	0	0	0	0	0	0	0	0	0	0	0	0	0	0	0	0	0	0	0	0	0	0	0	0
Increased Energy	0,60	-	0,35	0,14	0	0,37	0,16	0	0	0	0	0	0	0	0	0	0	0	0	0	0	0	0	0	0	0	0	0	0	0	0	0	0	0	0	0	0	0	0	0	0	0	0	0	0	0	0	0	0	0	0	0
Sexual Interest	0	0	-	0,11	0	0,16	0,07	0	0	0	0	0	0	0	0	0	0	0	0	0	0	0	0	0	0	0	0	0	0	0	0	0	0	0	0	0	0	0	0	0	0	0	0	0	0	0	0	0	0	0	0	0
Sleep	0,10	0,16	0	-	0	0,06	0,03	0	0	0	0	0	0	0	0,51	0	0	0	0	0	0	0	0	0	0	0	0	0	0	0	0	0	0	0	0	0	0	0	0	0	0,11	0	0	0	0	0	0	0	0	0	0	0
Irritability	0,06	0,12	0	0,02	-	0,02	0,04	0,02	0,24	0	0,14	0	0	0,28	0	0	0	0	0	0,02	0	0	0	0	0	0	0	0	0	0	0	0	0	0,01	0	0	0	0	0	0	0,02	0	0	0	0	0,05	0,01	0,01	0,09	0	0	0
Speech rate	0,42	0,49	0	0,06	0,06	-	0,40	0,17	0	0	0	0	0	0	0	0	0	0	0	0	0	0	0	0	0	0	0	0	0	0	0	0	0	0	0	0	0	0	0	0	0	0	0	0	0	0	0	0	0	0	0	0
Language-Thought Disorder	0,31	0,29	0	0,05	0,11	0,35	-	0,39	0,02	0	0	0	0	0,02	0	0	0	0	0	0	0	0	0	0	0	0	0	0	0	0	0	0	0	0	0	0	0	0	0	0	0	0	0	0	0	0	0	0	0,01	0	0	0
Content	0,08	0,11	0	0	0,05	0,12	0,47	-	0	0	0	0	0	0	0	0	0	0	0	0	0	0	0	0	0	0	0	0	0	0	0	0	0	0	0	0	0	0	0	0	0	0	0	0	0	0	0	0	0	0	0	0
Disruptive-Aggressive Behavior	0	0	0	0	0	0	0	0	-	0	0,60	0	0	0,08	0	0	0	0	0	0	0	0	0	0	0	0	0	0	0	0	0	0	0	0	0	0	0	0	0	0	0	0	0	0	0	0	0	0	0,03	0	0	0
Appearance	0	0	0	0	0	0	0	0,13	0	-	0	0	0	0	0	0	0	0	0	0	0	0	0	0	0	0	0	0	0	0	0	0	0	0	0	0	0	0	0	0	0	0	0	0	0	0	0	0	0	0	0	0
Insight	0	0	0	0	0	0	0,15	0,28	0	0,45	-	0	0	0	0	0	0	0	0	0	0	0	0	0	0	0	0	0	0	0	0	0	0	0	0	0	0	0	0	0	0	0	0	0	0	0	0	0	0	0	0	0
Apparent sadness	0	0	0	0	0	0	0	0	0	0	0	-	0,73	0	0	0	0	0	0	0	0	0	0	0	0	0	0	0	0	0	0	0	0	0	0	0	0	0	0	0	0	0	0	0	0	0	0	0	0	0	0	0
Reported sadness	0	0	0	0	0	0	0	0	0	0	0	0,72	-	0	0	0	0	0	0	0	0	0	0	0	0	0	0	0	0	0	0	0	0	0	0	0	0	0	0	0	0	0	0	0	0	0	0	0	0	0	0	0
Inner tension	0,03	0,07	0	0,01	0,25	0,02	0,04	0	0	0	0	0	0,02	-	0	0	0,00	0,01	0	0,06	0,03	0	0	0	0	0,01	0	0	0	0	0	0	0	0	0	0	0	0	0	0	0,02	0	0	0	0	0,12	0,03	0,05	0,06	0,01	0,01	0
Reduced sleep	0	0,10	0	0,60	0	0	0	0	0	0	0	0	0	0	-	0	0	0	0	0	0	0	0	0	0	0	0	0	0	0	0	0,01	0	0	0	0	0	0	0	0	0,21	0	0	0	0	0,03	0	0	0,03	0	0	0
Reduced appetite	0	0	0	0	0	0	0	0	0	0	0	0	0	0	0	-	0	0	0	0	0	0	0	0	0,14	0	0	0,02	0	0	0,02	0,05	0	0	0	0	0	0	0	0	0	0	0	0	0	0	0	0	0	0	0	0
Concentration difficulty	0	0	0	0	0	0	0	0	0	0	0	0	0	0	0	0	-	0,07	0,03	0,03	0,01	0	0	0	0	0	0	0	0	0	0	0	0	0	0	0	0	0	0	0	0	0,01	0	0	0	0,07	0,35	0,04	0,04	0	0,06	0
Lassitude	0	0	0	0	0	0	0	0	0	0	0	0	0	0	0	0	0,05	-	0,34	0,06	0,03	0	0	0	0	0	0	0	0	0	0	0	0	0	0	0	0	0	0	0	0	0,09	0	0	0	0,01	0,04	0,03	0	0,09	0,16	0,03
Inability to feel	0	0	0	0	0	0	0	0	0	0	0	0	0,02	0,01	0	0	0,01	0,27	-	0,02	0	0	0	0	0	0	0	0	0	0	0	0	0	0	0	0	0	0	0	0	0	0,02	0	0	0	0	0	0	0	0	0,06	0
Pessimistic thoughts	0	0,01	0	0	0,03	0	0	0	0	0	0	0,08	0,12	0,09	0	0	0	0,02	0,09	-	0,44	0	0	0	0	0	0	0	0	0	0	0	0	0	0	0	0	0	0	0	0	0,00	0	0	0	0,01	0,01	0	0	0	0,01	0
Suicidal thoughts	0	0	0	0	0	0	0	0	0	0	0	0	0,06	0,05	0	0	0	0	0,04	0,46	-	0	0	0	0	0	0	0	0	0	0	0	0	0	0	0	0	0	0	0	0	0	0	0	0	0	0	0	0	0	0	0
Diarrhea	0	0	0	0	0	0	0	0	0	0	0	0	0	0	0,01	0	0	0	0	0	0	-	0	0	0	0	0	0	0	0	0	0	0	0	0	0	0	0	0	0	0	0	0	0	0	0	0	0	0	0	0	0
Constipation	0	0	0	0	0	0	0	0	0	0	0	0	0	0	0	0	0	0	0	0	0	0	-	0,24	0	0	0	0	0,01	0	0,06	0,01	0,02	0	0	0,03	0	0	0	0,02	0	0	0	0	0	0	0	0	0	0	0	0,01
Dry mouth	0	0	0	0	0	0	0	0	0	0	0	0	0	0	0	0	0	0	0	0	0	0	0,05	-	0,04	0,00	0	0	0,06	0,10	0,18	0,05	0,07	0,05	0	0,10	0,03	0,01	0,01	0,08	0,00	0,00	0,00	0,00	0	0	0	0	0	0,01	0,00	0,04
Nausea and vomiting	0	0	0	0	0	0	0	0	0	0	0	0	0	0	0	0	0	0	0	0	0	0	0	0	-	0,05	0,03	0,09	0,02	0,09	0,10	0,29	0	0	0	0,01	0	0	0	0,02	0,01	0	0	0	0	0	0	0	0	0,02	0	0
Palpitations	0	0	0	0	0	0	0	0	0	0	0	0	0	0	0,01	0	0	0	0	0	0	0,05	0	0	0	-	0,24	0,44	0,11	0,02	0,01	0	0,01	0	0,11	0,01	0	0	0	0	0,01	0	0	0	0	0,06	0,02	0,03	0,01	0,02	0,00	0
Vertigo	0	0	0	0	0	0	0	0	0	0	0	0	0	0	0	0	0	0	0	0	0	0	0	0	0,21	0,24	-	0,27	0,03	0,01	0	0	0	0,19	0,68	0	0	0	0	0	0	0	0	0	0	0,01	0	0	0	0	0	0
Chest discomfort or pain	0	0	0	0	0	0	0	0	0	0	0	0	0	0	0	0	0	0	0	0	0	0	0	0,03	0	0,38	0,13	-	0,06	0,03	0,03	0,04	0	0	0,15	0	0,01	0	0	0	0	0	0	0	0	0,04	0	0	0	0	0	0
Sweating	0	0	0	0	0	0	0	0	0	0	0	0	0	0	0	0	0	0	0	0	0	0,21	0	0	0	0,21	0,08	0,09	-	0,12	0,15	0,02	0,04	0,03	0	0,05	0,02	0	0	0,02	0	0,02	0	0	0	0,02	0	0	0	0,10	0,06	0,00
Itching	0	0	0	0	0	0	0	0	0	0	0	0	0	0	0	0	0	0	0	0	0	0	0,12	0,14	0	0	0	0	0	-	0,52	0,05	0,00	0	0	0,02	0,10	0	0	0,10	0,00	0	0	0	0	0	0	0	0	0,01	0	0
Dry skin	0	0	0	0	0	0	0	0	0	0	0	0	0	0	0	0	0	0	0	0	0	0	0,20	0,25	0	0	0	0	0	0,71	-	0,08	0,03	0	0	0,03	0,10	0,02	0,02	0,15	0	0	0	0	0	0	0	0	0	0,01	0	0,01
Headache	0	0	0	0	0	0	0	0	0	0	0	0	0	0	0	0	0	0	0	0	0	0	0	0,10	0	0,13	0	0,32	0	0	0,02	-	0,01	0,00	0	0,03	0,01	0	0	0,00	0,03	0,01	0	0	0	0,01	0	0	0,01	0,06	0,04	0,00
Tremor	0	0	0	0	0	0	0	0	0	0	0	0	0	0	0	0	0	0	0	0	0	0	0	0	0	0,10	0,04	0,05	0,03	0	0	0	-	0,54	0,16	0,07	0	0	0	0,01	0	0	0	0	0	0	0	0	0,04	0,01	0	0,01
Impaired motor control	0	0	0	0	0	0	0	0	0	0	0	0	0	0	0	0	0	0	0	0	0	0	0	0	0,10	0,05	0,23	0	0	0	0	0	0,41	-	0,24	0,09	0,02	0	0	0	0,01	0	0	0	0	0,01	0,01	0,01	0,07	0	0	0
Dizziness	0	0,01	0	0	0,01	0	0	0	0	0	0	0	0	0,01	0	0	0	0	0	0	0	0	0	0	0,27	0,19	0,68	0	0	0	0	0	0,14	0,31	-	0,04	0	0	0	0	0	0	0	0	0	0	0	0	0,03	0	0	0
Blurred vision	0	0	0	0	0	0	0	0	0	0	0	0	0	0	0	0	0	0	0	0	0	0,07	0	0	0	0	0	0	0,02	0	0	0	0,03	0	0	-	0,13	0	0	0,04	0,00	0	0	0	0	0	0	0	0,01	0,01	0	0,01
Tinnitus	0	0	0	0	0	0	0	0	0	0	0	0	0	0	0	0	0	0	0	0	0	0	0,03	0,04	0	0	0	0	0	0,11	0,18	0	0	0	0	0	-	0,03	0,03	0,19	0	0	0	0	0	0	0	0	0	0	0	0
Difficulty urinating	0	0	0	0	0	0	0	0	0	0	0	0	0	0	0	0	0	0	0	0	0	0	0	0	0	0	0	0	0	0	0	0	0,02	0	0	0,04	0	-	0,98	0,21	0	0	0	0	0	0	0	0	0	0	0	0
Painful urination	0	0	0	0	0	0	0	0	0	0	0	0	0	0	0	0	0	0	0	0	0	0	0	0	0	0	0	0	0	0	0	0	0,06	0	0	0,11	0	0,94	-	0,21	0	0	0	0	0	0	0	0	0	0	0	0
Frequent urination	0	0	0	0	0	0	0	0	0	0	0	0	0	0	0	0	0	0	0	0	0	0,01	0	0	0	0,01	0	0	0	0	0	0	0,10	0,05	0	0,19	0	0,46	0,58	-	0	0	0	0	0	0	0	0	0	0	0	0,00
Trouble falling asleep	0	0	0	0,07	0	0	0	0	0	0	0	0	0	0,03	0,11	0	0,02	0	0	0	0	0,08	0	0	0	0,07	0,03	0,03	0,04	0	0	0	0,01	0	0	0,01	0	0	0	0	-	0	0	0	0	0,16	0,05	0,07	0,15	0,01	0,01	0
Increased sleep time	0	0	0	0	0	0	0	0	0	0	0	0	0	0	0	0	0	0	0	0	0	0	0	0	0	0	0	0	0	0	0	0	0	0	0	0	0	0	0	0	0	-	0,00	0	0	0,02	0,02	0,03	0	0,22	0,25	0,09
Reduced sex drive	0	0	0	0	0	0	0	0	0	0	0	0	0	0	0	0	0	0	0	0	0	0	0	0	0	0	0	0	0	0	0	0	0	0	0	0	0	0	0	0	0	0	-	0,84	0	0	0	0	0	0	0,01	0,06
Orgasm disorders	0	0	0	0	0	0	0	0	0	0	0	0	0	0	0	0	0	0	0	0	0	0	0	0	0	0	0	0	0	0	0	0	0	0	0	0	0	0	0	0	0	0	0,56	-	0	0	0	0	0	0	0,01	0,04
Erectile Dysfunction / Irregular periods	0	0	0	0	0	0	0	0	0	0	0	0	0	0	0	0	0	0	0	0	0	0	0	0	0	0	0	0	0	0	0	0	0	0	0	0	0	0	0	0	0	0	0,51	0,66	-	0	0	0	0	0	0	0
Anxiety	0	0,02	0	0	0,05	0	0	0	0	0	0	0	0	0,13	0,02	0	0,07	0	0	0,02	0	0,02	0	0	0	0,01	0	0	0	0	0	0	0	0	0,01	0	0	0	0	0	0,18	0,01	0	0	0	-	0,20	0,34	0,22	0,06	0,10	0,01
Impaired concentration	0	0,02	0	0	0,02	0	0	0	0	0	0	0	0	0,05	0,01	0	0,31	0,01	0	0	0	0,01	0	0	0	0,00	0	0	0	0	0	0	0	0	0,02	0	0	0	0	0	0,09	0,01	0	0	0	0,22	-	0,19	0,13	0,06	0,11	0,02
General malaise	0	0,01	0	0	0,01	0	0	0	0	0	0	0	0	0,06	0	0	0,05	0	0	0	0	0	0	0	0	0	0	0	0	0	0	0	0	0	0,01	0	0	0	0	0	0,09	0	0	0	0	0,41	0,24	-	0,17	0,09	0,15	0,02
Agitation	0,05	0,09	0	0,02	0,10	0,03	0,03	0	0	0	0	0	0	0,10	0	0	0,06	0	0	0,01	0	0	0	0	0,04	0	0,08	0	0	0	0	0	0	0,04	0,09	0	0	0	0	0	0,04	0,02	0	0	0	0,17	0,17	0,13	-	0,01	0,04	0
Asthenia	0	0,01	0	0	0,01	0	0	0	0	0	0	0	0	0,01	0	0	0,02	0	0	0	0	0	0	0	0	0	0	0	0	0	0	0	0	0	0,01	0	0	0	0	0	0,01	0,18	0	0	0	0,05	0,14	0,09	0,08	-	0,46	0,09
Loss of energy	0	0	0	0	0	0	0	0	0	0	0	0	0	0	0	0	0,06	0	0	0	0	0	0	0	0	0	0	0	0	0	0	0	0	0	0	0	0	0	0	0	0,01	0,09	0	0	0	0,11	0,18	0,15	0,07	0,43	-	0,14
Weight gain	0	0	0	0	0	0	0	0	0	0	0	0	0	0	0	0	0	0	0	0	0	0	0	0	0	0	0	0	0	0	0	0	0	0	0	0	0	0	0	0	0	0,15	0	0	0	0	0	0	0	0,03	0	-

Supplementary Table 4. Predictability of each node in the two networks. The sex differences in predictability that can be considered moderate (> 0.15) are in bold.

	Proportion of explained variance (R^2)		Difference between males and females
Nodes	Males	Females	
PRISM08B: Orgasm disorders	0.459	0.414	- 0.045
PRISM09E: Asthenia	0.453	0.510	0.057
PRISM09F: Loss of energy	0.444	0.558	0.114
PRISM08C: Erectile dysfunction	0.437		-
PRISM06D: Irregular periods		0	-
PRISM09C: General malaise	0.427	0.370	- 0.273
PRISM09B: Impaired concentration	0.419	0.392	- 0.027
PRISM09A: Anxiety	0.379	0.380	0.001
PRISM08A: Reduced sex drive	0.374	0.432	0.058
YMRS04: Sleep	0.32	0.34	0.02
YMRS02: Increased Energy	0.311	0.283	- 0.028
MADRS04: Reduced sleep	0.303	0.266	- 0.037
PRISM09D: Agitation	0.289	0.308	0.019
YMRS01: Elevated Mood	0.258	0.319	0.061
PRISM02B: Vertigo	0.251	0.263	0.012
PRISM06B: Painful urination	0.246	0.075	**- 0.171**
MADRS02: Reported sadness	0.245	0.232	- 0.013
PRISM06A: Difficulty urinating	0.240	0.127	- 0.113
PRISM04D: Dizziness	0.239	0.256	0.017
PRISM07A: Trouble falling asleep	0.229	0.197	- 0.032
YMRS06: Speech rate	0.221	0.264	0.043
PRISM02A: Palpitations	0.213	0.183	- 0.03
PRISM03C: Dry skin	0.209	0.268	0.059
YMRS07: Language−Thought Disorder	0.201	0.198	- 0.003
MADRS03: Inner tension	0.190	0.228	0.038
MADRS09: Pessimistic thoughts	0.170	0.178	0.008
PRISM07B: Increased sleep time	0.156	0.21	0.054
PRISM04C: Impaired motor control	0.147	0.251	0.104

PRISM09G: Weight gain	0.146	0.138	- 0.008
YMRS05: Irritability	0.143	0.176	0.033
MADRS01: Apparent sadness	0.137	0.288	**0.151**
MADRS06: Concentration difficulty	0.128	0.232	0.104
PRISM06C: Frequent urination	0.124	0.123	- 0.001
PRISM04B: Tremor	0.120	0.174	0.054
MADRS07: Lassitude	0.118	0.244	0.126
PRISM05A: Blurred vision	0.116	0.172	0.056
PRISM03B: Itching	0.115	0.292	**0.177**
MADRS08: Inability to feel	0.11	0.145	0.035
PRISM02C: Chest discomfort or pain	0.096	0.186	0.09
PRISM03A: Increased sweating	0.095	0.147	0.052
YMRS08: Content	0.091	0.065	- 0.026
PRISM01A: Diarrhea	0.086	0	- 0.086
MADRS10: Suicidal thoughts	0.085	0.170	0.085
PRISM04A: Headache	0.083	0.152	0.069
PRISM01B: Constipation	0.043	0.051	0.008
YMRS03: Sexual Interest	0.036	0.066	0.03
PRISM01C: Dry mouth	0.036	0.197	**0.161**
PRISM05B: Tinnitus	0.031	0.086	0.055
YMRS11: Insight	0.026	0.012	- 0.014
PRISM01D: Nausea and vomiting	0.019	0.192	**0.173**
YMRS10: Appearance	0.014	0	- 0.014
YMRS09: Disruptive−Aggressive Behavior	0	0.07	0.07
MADRS05: Reduced appetite	0	0.005	0.005

Supplementary Table 5. Stabilizing index of each node in the two estimated networks. The stabilizing index is the sum of absolute edge weight values a given node has within its community.

	Males		Females	
	**Cluster**	**Stabilizing index (raw value)**	**Cluster**	**Stabilizing index (raw value)**
YMRS02: Increased Energy	1 (mood)	2.13	1 (mood)	1.24
YMRS07: Language−Thought Disorder	1 (mood)	1.77	1 (mood)	1.25
PRISM04D: Dizziness	2 (non-sexual side effects)	1.74	2 (non-sexual side effects)	1.43
PRISM06B: Painful urination	2 (non-sexual side effects)	1.69	2 (non-sexual side effects)	1.19
PRISM09A: Anxiety	1 (mood)	1.55	1 (mood)	1.46
PRISM09B: Impaired concentration	1 (mood)	1.47	1 (mood)	1.16
PRISM02B: Vertigo	2 (non-sexual side effects)	1.47	2 (non-sexual side effects)	1.56
PRISM02A: Palpitations	2 (non-sexual side effects)	1.43	2 (non-sexual side effects)	1.03
PRISM03C: Dry skin	2 (non-sexual side effects)	1.37	2 (non-sexual side effects)	1.56
YMRS01: Elevated Mood	1 (mood)	1.32	1 (mood)	2.45
PRISM06C: Frequent urination	2 (non-sexual side effects)	1.30	2 (non-sexual side effects)	0.92
PRISM06A: Difficulty urinating	2 (non-sexual side effects)	1.23	2 (non-sexual side effects)	1.16
PRISM08B: Orgasm disorders	3 (sexual side effects)	1.22	3 (sexual side effects)	0.84
YMRS06: Speech rate	1 (mood)	1.20	1 (mood)	1.33
PRISM08C: Erectile dysfunction	3 (sexual side effects)	1.14		
PRISM09E: Asthenia	1 (mood)	1.08	1 (mood)	0.93
YMRS08: Content	1 (mood)	1.07	1 (mood)	0.39
PRISM09F: Loss of energy	1 (mood)	1.07	1 (mood)	1.69
PRISM09C: General malaise	1 (mood)	1.06	1 (mood)	0.97
PRISM04C: Impaired motor control	2 (non-sexual side effects)	1.02	2 (non-sexual side effects)	1.22
PRISM09D: Agitation	1 (mood)	1.01	1 (mood)	0.92
YMRS04: Sleep	1 (mood)	0.98	1 (mood)	0.95
MADRS09: Pessimistic thoughts	1 (mood)	0.93	1 (mood)	0.66
MADRS02: Reported sadness	1 (mood)	0.91	4 (reported and apparent sadness	0.73
PRISM08A: Reduced sex drive	3 (sexual side effects)	0.90	3 (sexual side effects)	0.84
PRISM04B: Tremor	2 (non-sexual side effects)	0.90	2 (non-sexual side effects)	0.83

PRISM02C: Chest discomfort or pain	2 (non-sexual side effects)	0.88	2 (non-sexual side effects)	1.14
MADRS04: Reduced sleep	1 (mood)	0.87	1 (mood)	0.96
YMRS11: Insight	1 (mood)	0.86	1 (mood)	0.60
MADRS03: Inner tension	1 (mood)	0.83	1 (mood)	0.63
MADRS01: Apparent sadness	1 (mood)	0.72	4 (reported and apparent sadness)	0.73
PRISM03B: Itching	2 (non-sexual side effects)	0.71	2 (non-sexual side effects)	1.07
YMRS05: Irritability	1 (mood)	0.65	1 (mood)	0.98
PRISM03A: Increased sweating	2 (non-sexual side effects)	0.51	2 (non-sexual side effects)	0.62
PRISM04A: Headache	2 (non-sexual side effects)	0.48	2 (non-sexual side effects)	0.59
PRISM07A: Trouble falling asleep	1 (mood)	0.48	1 (mood)	0.69
MADRS10: Suicidal thoughts	1 (mood)	0.46	1 (mood)	0.44
YMRS10: Appearance	1 (mood)	0.45	one-node cluster	0
PRISM01C: Dry mouth	2 (non-sexual side effects)	0.41	2 (non-sexual side effects)	0.99
MADRS08: Inability to feel	1 (mood)	0.40	1 (mood)	0.34
MADRS06: Concentration difficulty	1 (mood)	0.39	1 (mood)	0.54
PRISM07B: Increased sleep time	1 (mood)	0.36	1 (mood)	0.53
MADRS07: Lassitude	1 (mood)	0.34	1 (mood)	0.85
PRISM01A: Diarrhea	2 (non-sexual side effects)	0.33	one-node cluster	0
PRISM05A: Blurred vision	2 (non-sexual side effects)	0.28	2 (non-sexual side effects)	0.56
PRISM01D: Nausea and vomiting	2 (non-sexual side effects)	0.27	2 (non-sexual side effects)	0.85
PRISM01B: Constipation	2 (non-sexual side effects)	0.20	2 (non-sexual side effects)	0.24
PRISM05B: Tinnitus	2 (non-sexual side effects)	0.18	2 (non-sexual side effects)	0.58
PRISM09G: Weight gain	1 (mood)	0.15	1 (mood)	0.25
YMRS03: Sexual Interest	one-node cluster	0	1 (mood)	0.61
YMRS09: Disruptive-Aggressive Behavior	one-node cluster	0	1 (mood)	0.98
MADRS05: Reduced appetite	one-node cluster	0	2 (non-sexual side effects)	0.14
PRISM06D: Irregular periods			one-node cluster	0

Supplementary Figure 1. Betweenness and closeness of each node of the network. a: females, **b**: males. Each line corresponds to one item of the YMRS, MADRS, or PRISE-M questionnaire. Strength is reported as raw scores. Centrality indices were obtained using the *centralityPlot* function of the package *bootnet* (see supplementary information).

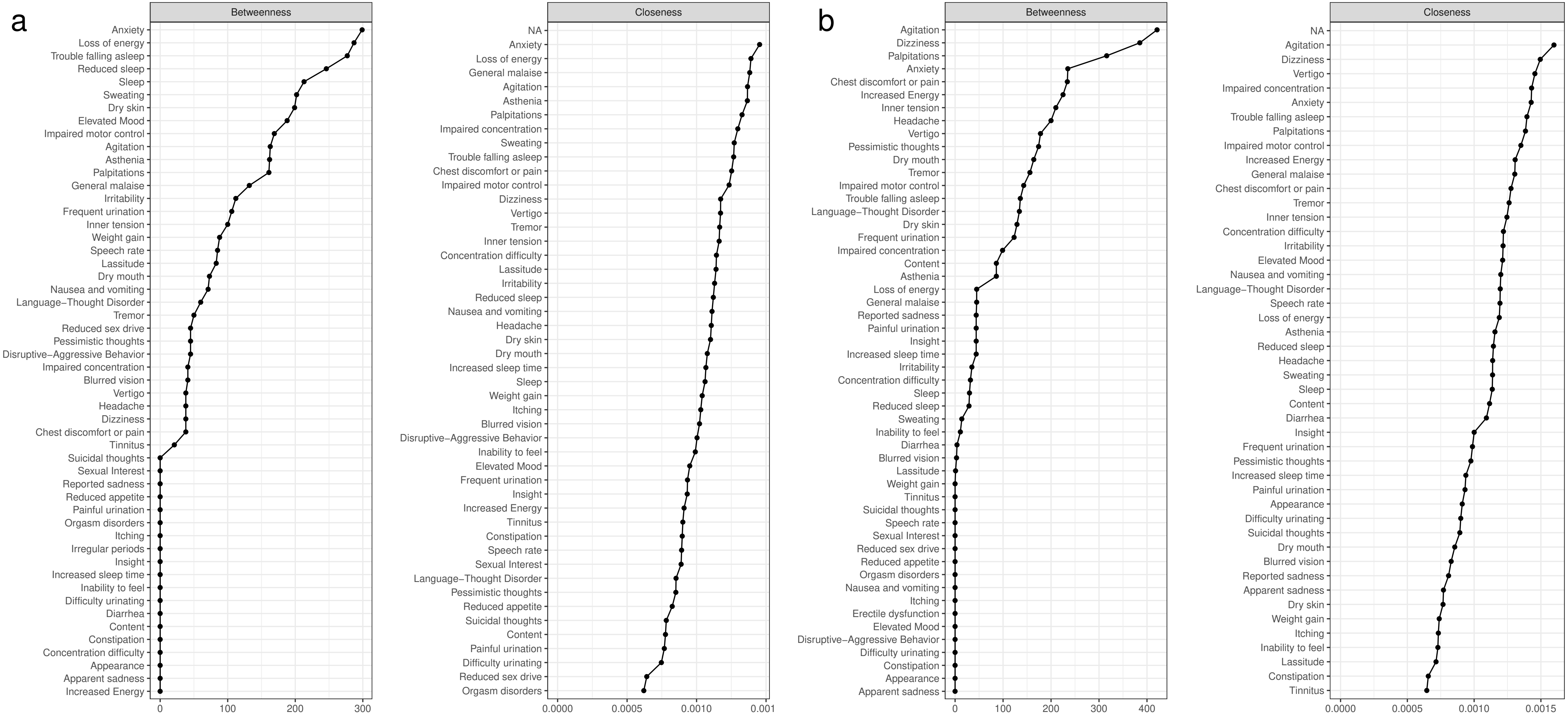
a
Betweenness
Anxiety
Loss of energy
Trouble falling asleep
Reduced sleep
Sleep
Sweating
Dry skin
Elevated Mood
Impaired motor control
Agitation
Asthenia
Palpitations
General malaise
Irritability
Frequent urination
Inner tension
Weight gain
Speech rate
Lassitude
Dry mouth
Nausea and vomiting
Language−Thought Disorder
Tremor
Reduced sex drive
Pessimistic thoughts
Disruptive−Aggressive Behavior
Impaired concentration
Blurred vision
Vertigo
Headache
Dizziness
Chest discomfort or pain
Tinnitus
Suicidal thoughts
Sexual Interest
Reported sadness
Reduced appetite
Painful urination
Orgasm disorders
Itching
Irregular periods
Insight
Increased sleep time
Inability to feel
Difficulty urinating
Diarrhea
Content
Constipation
Concentration difficulty
Appearance
Apparent sadness
Increased Energy
0
100
200
300
Closeness
NA
Anxiety
Loss of energy
General malaise
Agitation
Asthenia
Palpitations
Impaired concentration
Sweating
Trouble falling asleep
Chest discomfort or pain
Impaired motor control
Dizziness
Vertigo
Tremor
Inner tension
Concentration difficulty
Lassitude
Irritability
Reduced sleep
Nausea and vomiting
Headache
Dry skin
Dry mouth
Increased sleep time
Sleep
Weight gain
Itching
Blurred vision
Disruptive−Aggressive Behavior
Inability to feel
Elevated Mood
Frequent urination
Insight
Increased Energy
Tinnitus
Constipation
Speech rate
Sexual Interest
Language−Thought Disorder
Pessimistic thoughts
Reduced appetite
Suicidal thoughts
Content
Painful urination
Difficulty urinating
Reduced sex drive
Orgasm disorders
0.0000
0.0005
0.0010
0.001
b
Betweenness
Agitation
Dizziness
Palpitations
Anxiety
Chest discomfort or pain
Increased Energy
Inner tension
Headache
Vertigo
Pessimistic thoughts
Dry mouth
Tremor
Impaired motor control
Trouble falling asleep
Language−Thought Disorder
Dry skin
Frequent urination
Impaired concentration
Content
Asthenia
Loss of energy
General malaise
Reported sadness
Painful urination
Insight
Increased sleep time
Irritability
Concentration difficulty
Sleep
Reduced sleep
Sweating
Inability to feel
Diarrhea
Blurred vision
Lassitude
Weight gain
Tinnitus
Suicidal thoughts
Speech rate
Sexual Interest
Reduced sex drive
Reduced appetite
Orgasm disorders
Nausea and vomiting
Itching
Erectile dysfunction
Elevated Mood
Disruptive−Aggressive Behavior
Difficulty urinating
Constipation
Appearance
Apparent sadness
0
100
200
300
400
Closeness
NA
Agitation
Dizziness
Vertigo
Impaired concentration
Anxiety
Trouble falling asleep
Palpitations
Impaired motor control
Increased Energy
General malaise
Chest discomfort or pain
Tremor
Inner tension
Concentration difficulty
Irritability
Elevated Mood
Nausea and vomiting
Language−Thought Disorder
Speech rate
Loss of energy
Asthenia
Reduced sleep
Headache
Sweating
Sleep
Content
Diarrhea
Insight
Frequent urination
Pessimistic thoughts
Increased sleep time
Painful urination
Appearance
Difficulty urinating
Suicidal thoughts
Dry mouth
Blurred vision
Reported sadness
Apparent sadness
Dry skin
Weight gain
Itching
Inability to feel
Lassitude
Constipation
Tinnitus
0.0000
0.0005
0.0010
0.0015

Supplementary Figure 2. CS-coefficients for each proportion of the original sample excluded from the case-dropping bootstrap samples (2500 iterations). a: females, **b**: males. The CS-coefficient is the correlation coefficient between centrality indices in the original sample and the bootstrapped samples.

a

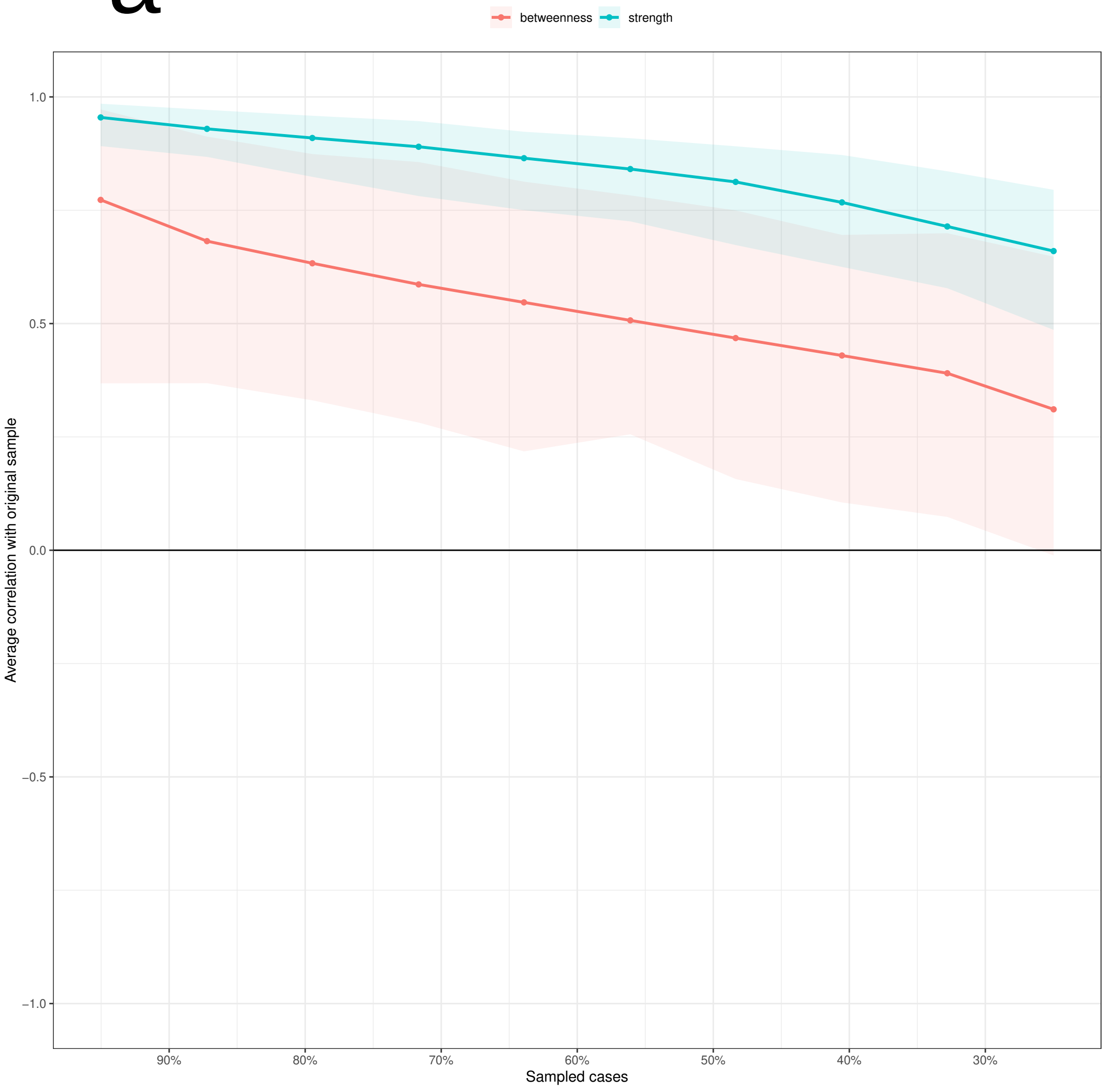
betweenness
strength
1.0
0.5
0.0
−0.5
−1.0
Average correlation with original sample
90%
80%
70%
60%
50%
40%
30%
Sampled cases

b

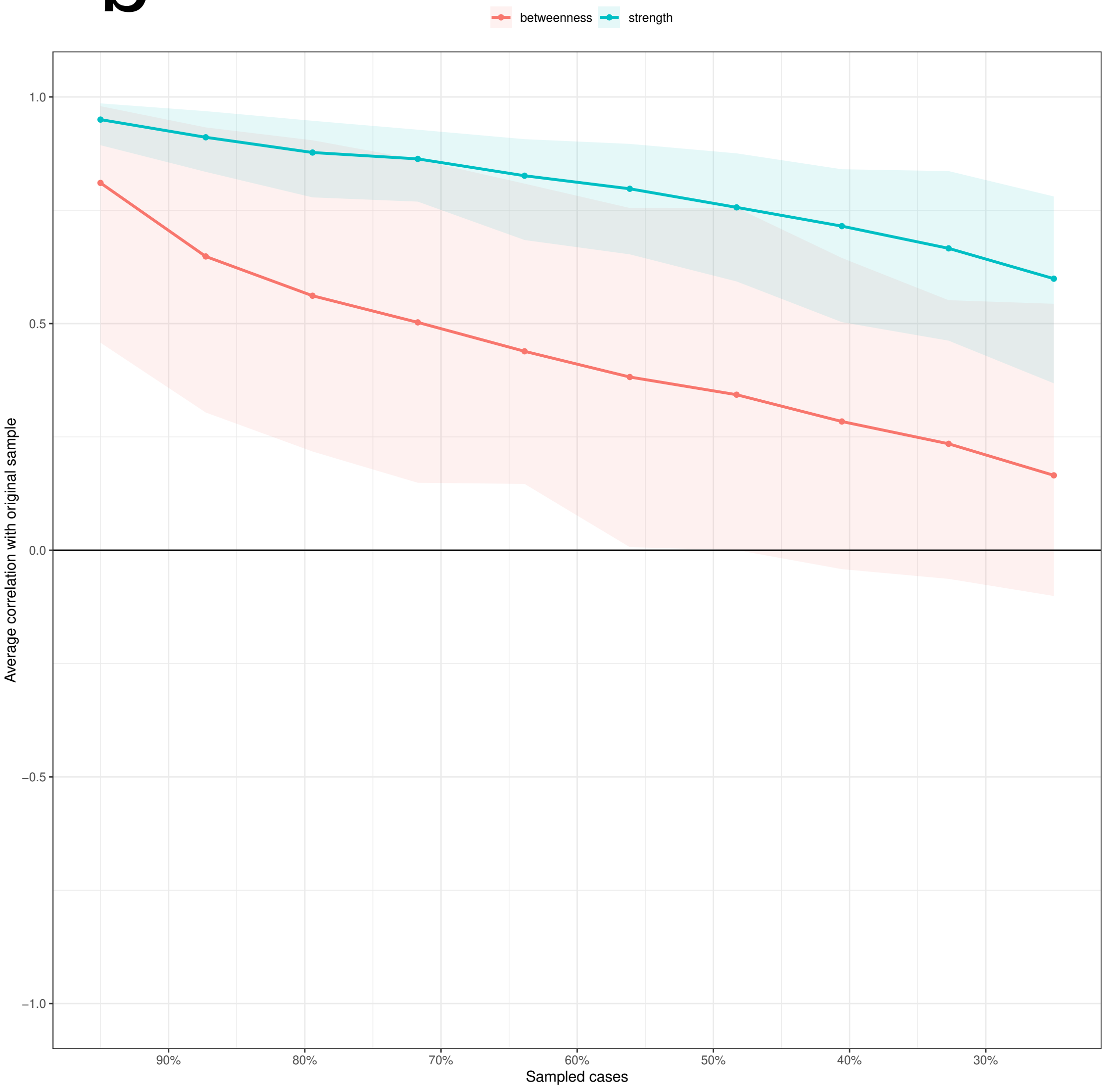
betweenness
strength
1.0
0.5
0.0
−0.5
−1.0
Average correlation with original sample
90%
80%
70%
60%
50%
40%
30%
Sampled cases

Supplementary Figure 3. Bootstrapped difference tests (α = 0.05) between values of node strength for each pair of nodes. a: females, **b**: males. Numbers on the diagonal are the node strengths in the original sample. Gray boxes indicate strength values that do not significantly differ from one another and black boxes represent strength values that do. For example, tinnitus was a significantly weaker node than painful urination.

a

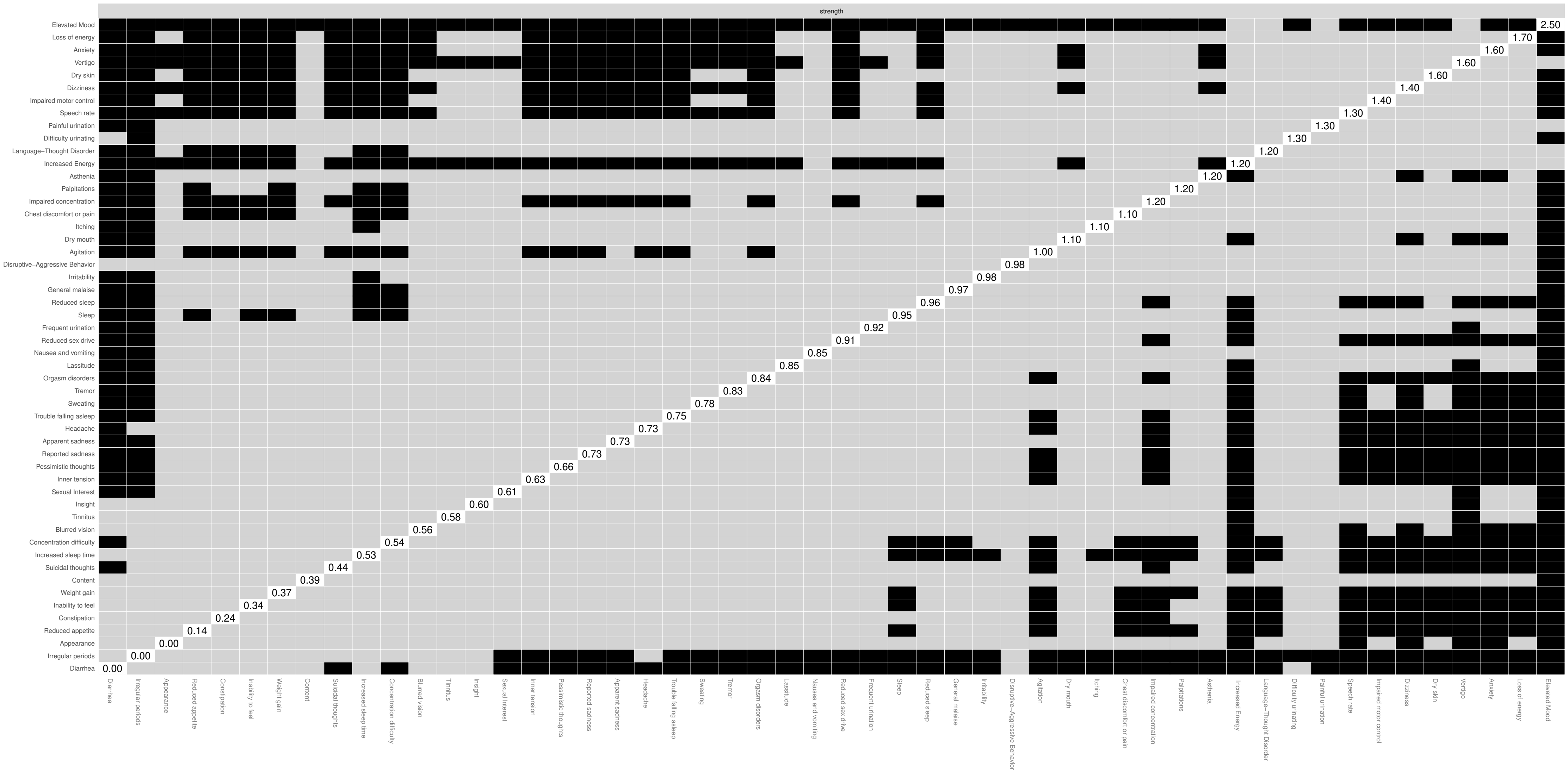
strength
Elevated Mood
Loss of energy
Anxiety
Vertigo
Dry skin
Dizziness
Impaired motor control
Speech rate
Painful urination
Difficulty urinating
Language−Thought Disorder
Increased Energy
Asthenia
Palpitations
Impaired concentration
Chest discomfort or pain
Itching
Dry mouth
Agitation
Disruptive−Aggressive Behavior
Irritability
General malaise
Reduced sleep
Sleep
Frequent urination
Reduced sex drive
Nausea and vomiting
Lassitude
Orgasm disorders
Tremor
Sweating
Trouble falling asleep
Headache
Apparent sadness
Reported sadness
Pessimistic thoughts
Inner tension
Sexual Interest
Insight
Tinnitus
Blurred vision
Concentration difficulty
Increased sleep time
Suicidal thoughts
Content
Weight gain
Inability to feel
Constipation
Reduced appetite
Appearance
Irregular periods
Diarrhea
2.50
1.70
1.60
1.60
1.60
1.40
1.40
1.30
1.30
1.30
1.20
1.20
1.20
1.20
1.20
1.10
1.10
1.10
1.00
0.98
0.98
0.97
0.96
0.95
0.92
0.91
0.85
0.85
0.84
0.83
0.78
0.75
0.73
0.73
0.73
0.66
0.63
0.61
0.60
0.58
0.56
0.54
0.53
0.44
0.39
0.37
0.34
0.24
0.14
0.00
0.00
0.00

b

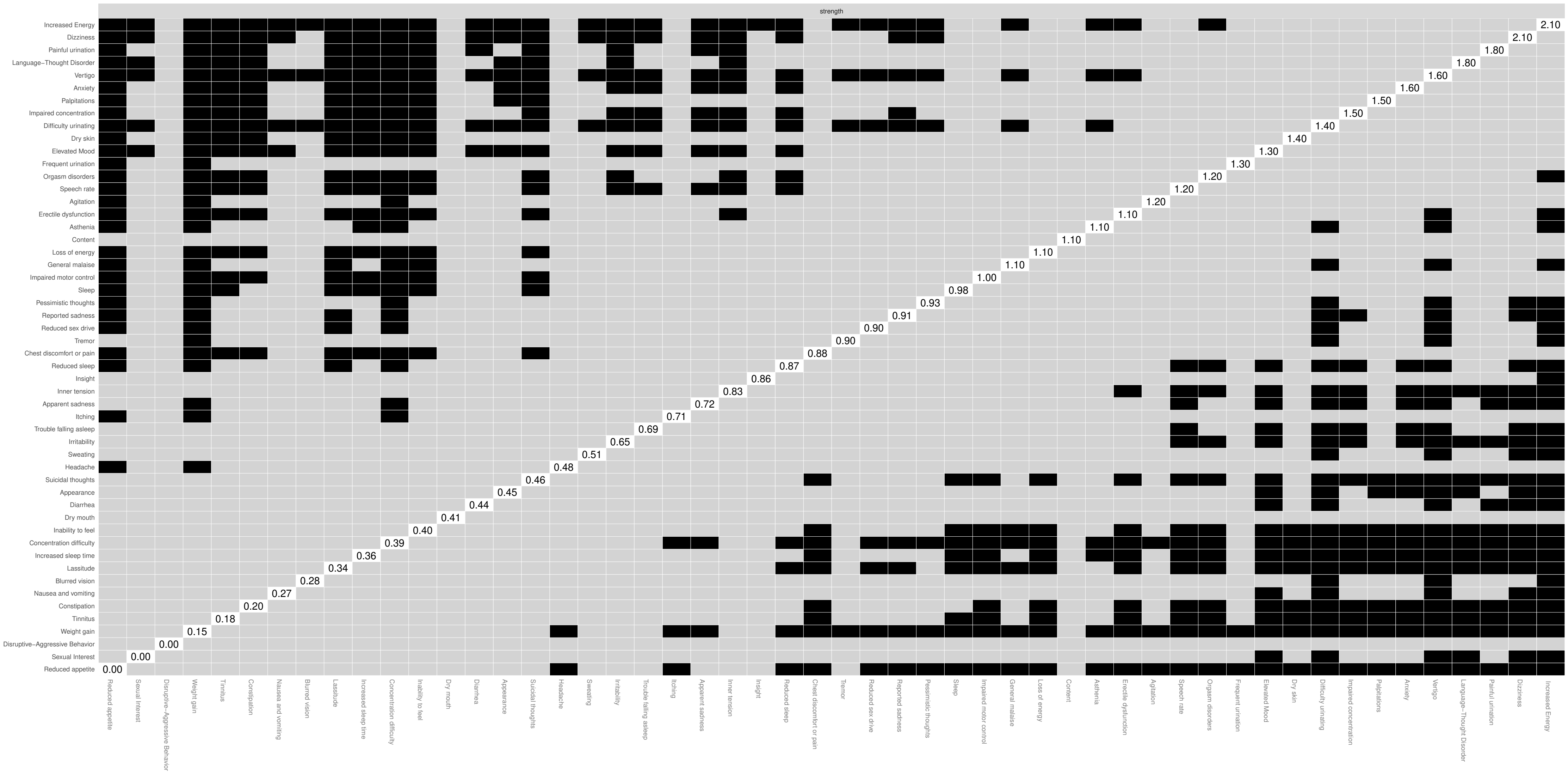
strength
Increased Energy
Dizziness
Painful urination
Language–Thought Disorder
Vertigo
Anxiety
Palpitations
Impaired concentration
Difficulty urinating
Dry skin
Elevated Mood
Frequent urination
Orgasm disorders
Speech rate
Agitation
Erectile dysfunction
Asthenia
Content
Loss of energy
General malaise
Impaired motor control
Sleep
Pessimistic thoughts
Reported sadness
Reduced sex drive
Tremor
Chest discomfort or pain
Reduced sleep
Insight
Inner tension
Apparent sadness
Itching
Trouble falling asleep
Irritability
Sweating
Headache
Suicidal thoughts
Appearance
Diarrhea
Dry mouth
Inability to feel
Concentration difficulty
Increased sleep time
Lassitude
Blurred vision
Nausea and vomiting
Constipation
Tinnitus
Weight gain
Disruptive–Aggressive Behavior
Sexual Interest
Reduced appetite
2.10
2.10
1.80
1.80
1.60
1.60
1.50
1.50
1.40
1.40
1.30
1.30
1.20
1.20
1.20
1.10
1.10
1.10
1.10
1.10
1.00
0.98
0.93
0.91
0.90
0.90
0.88
0.87
0.86
0.83
0.72
0.71
0.69
0.65
0.51
0.48
0.46
0.45
0.44
0.41
0.40
0.39
0.36
0.34
0.28
0.27
0.20
0.18
0.15
0.00
0.00
0.00

Supplementary Figure 4. Bridge strength of each node in the two networks. a: females, **b**: males. Each line corresponds to one item of the YMRS, MADRS or PRISE-M. Centrality indices were reported as raw scores. Bridge strength was measured with the *bridge* function of the R package *networktools* (see Supplementary Information).

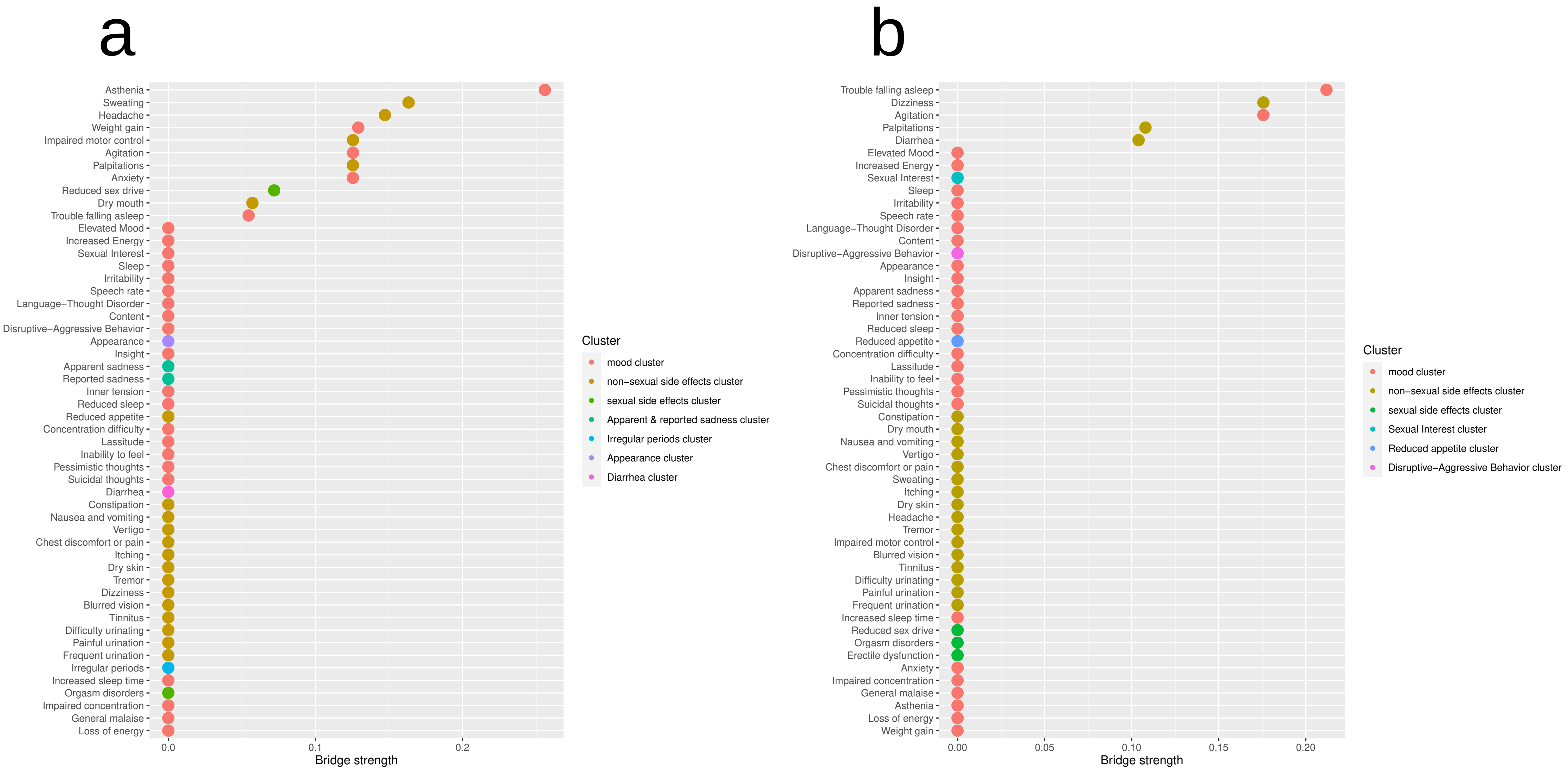
a
b
Asthenia
Sweating
Headache
Weight gain
Impaired motor control
Agitation
Palpitations
Anxiety
Reduced sex drive
Dry mouth
Trouble falling asleep
Elevated Mood
Increased Energy
Sexual Interest
Sleep
Irritability
Speech rate
Language−Thought Disorder
Content
Disruptive−Aggressive Behavior
Appearance
Insight
Apparent sadness
Reported sadness
Inner tension
Reduced sleep
Reduced appetite
Concentration difficulty
Lassitude
Inability to feel
Pessimistic thoughts
Suicidal thoughts
Diarrhea
Constipation
Nausea and vomiting
Vertigo
Chest discomfort or pain
Itching
Dry skin
Tremor
Dizziness
Blurred vision
Tinnitus
Difficulty urinating
Painful urination
Frequent urination
Irregular periods
Increased sleep time
Orgasm disorders
Impaired concentration
General malaise
Loss of energy
0.0
0.1
0.2
Bridge strength
Cluster
mood cluster
non−sexual side effects cluster
sexual side effects cluster
Apparent & reported sadness cluster
Irregular periods cluster
Appearance cluster
Diarrhea cluster
Trouble falling asleep
Dizziness
Agitation
Palpitations
Diarrhea
Elevated Mood
Increased Energy
Sexual Interest
Sleep
Irritability
Speech rate
Language−Thought Disorder
Content
Disruptive−Aggressive Behavior
Appearance
Insight
Apparent sadness
Reported sadness
Inner tension
Reduced sleep
Reduced appetite
Concentration difficulty
Lassitude
Inability to feel
Pessimistic thoughts
Suicidal thoughts
Constipation
Dry mouth
Nausea and vomiting
Vertigo
Chest discomfort or pain
Sweating
Itching
Dry skin
Headache
Tremor
Impaired motor control
Blurred vision
Tinnitus
Difficulty urinating
Painful urination
Frequent urination
Increased sleep time
Reduced sex drive
Orgasm disorders
Erectile dysfunction
Anxiety
Impaired concentration
General malaise
Asthenia
Loss of energy
Weight gain
0.00
0.05
0.10
0.15
0.20
Bridge strength
Cluster
mood cluster
non−sexual side effects cluster
sexual side effects cluster
Sexual Interest cluster
Reduced appetite cluster
Disruptive−Aggressive Behavior cluster
